# Supplementary material for: Chalcone synthase (CHS) family members analysis from eggplant (Solanum melongena L.) in the flavonoid biosynthetic pathway and expression patterns in response to heat stress
Source: PLoS One. 2020 Apr 17;15(4):e0226537. doi: 10.1371/journal.pone.0226537 (PMC7164647; doi:10.1371/journal.pone.0226537)
Supplement: S3 Table — (DOCX) [file pone.0226537.s003.docx]

**S3 Table Primers used for real time PCR analysis**

| Genes ID | Name |  | **Sequence 5'-3'** | **Reference** |
| --- | --- | --- | --- | --- |
| Sme2.5_01077.1_g00016.1 | *SmCHS1* | F | ATTGGCACCACAACTCCTTC |  |
|  |  | R | GGCTGCTTCTTTACCAAGTT |  |
| Sme2.5_02154.1_g00001.1 | *SmCHS2* | F | AGCACAGCGACTCCTTCAA |  |
|  |  | R | GGCTGCTTCTTTACCAAGTT |  |
| Sme2.5_13923.1_g00001.1 | *SmCHS3* | F | AAGGACTTGGCTGAGAACA |  |
|  |  | R | CAACACCTGAAATTGGGTC |  |
| Sme2.5_01039.1_g00002.1 | *SmCHS5* | F | GGCACTGTTCTCCGATTAGC |  |
|  |  | R | CGTCAGTCTCGGTCTCACTT |  |
| Sme2.5_00346.1_g00019.1 | *SmCHS6* | F | TTGGGATGTTATGGTGGTGT |  |
|  |  | R | GTCTAGCATTATTCGGTGGC |  |
| Sme2.5_05261.1_g00004.1 | *SmCHS7* | F | TTCAAACCACCAAGTGTCG |  |
|  |  | R | TGAATAGCAGTATGCAATTCA |  |
| Sme2.5_16832.1_g00001.1 |  | F | GAGGCTGCTGCTATTATGGA |  |
|  |  | R | TGGATTGTCATTCACCGAGT |  |
| Sme2.5_04410.1_g00008.1 |  | F | CTTTAGTGCCGCCGATTCTC |  |
|  |  | R | ATTCCGTCAACCCATACCCT |  |
| Sme2.5_00228.1_g00013.1 |  | F | GGTCGATAGCAGAAGCCTTAG |  |
|  |  | R | CAACAGACTTATGTGCCAAGAT |  |
| Sme2.5_00537.1_g00012.1 |  | F | TGAAAACAAAATCAATCGTCA |  |
|  |  | R | GAGTCCAACTTCAGCATCAC |  |
| Sme2.5_29845.1_g00001.1 |  | F | CTGTGCAGCCAATAGAAGTTA |  |
|  |  | R | CATTCGGATTCCGTCAAGTC |  |
| Sme2.5_00283.1_g00002.1 |  | F | GGAGAGCTGCTTGATAAAGG | [34] |
|  |  | R | AGAATCCAGAACATTGAGTTCC |  |
| Sme2.5_00188.1_g00020.1 |  | F | ATCGTTCGCGATAGAAGGTAA | [34] |
|  |  | R | CCTACTTCCACCTAAGTACCAT |  |
| Sme2.5_00015.1_g00020.1 |  | F | CATTGTCTCTAGCCATCTACAG | [34] |
|  |  | R | GAAGTAGGTCACTATTTCACGC |  |
| Sme2.5_04313.1_g00001.1 |  | F | TATGGCCTATAATGCCCAAGAC | [34] |
|  |  | R | TTCCTTAGCAACTTCCAACGA |  |
| Sme2.5_01193.1_g00009.1 |  | F | GAGTGAGACCTTCCCACA | [34] |
|  |  | R | GCATTACCAATGCTAGGGAC |  |
| Sme2.5_29581.1_g00001.1 |  | F | TCAGGAACAGACGTGATCG | [34] |
|  |  | R | TCATCCCTTCCATCACCCA |  |
| Sme2.5_03336.1_g00008.1 | *SmPAL* | F | TGCCTATGCTGATGATCCCT | [43] |
|  |  | R | GTTCCCACTTTCCAACGCTA |  |
| Sme2.5_01401.1_g00004.1 | *SmDFR* | F | TTCATTTGCTCATCCCATC | [43] |
|  |  | R | GCCCCTTGATACATATCCTC |  |
| Sme2.5_00747.1_g00012.1 | *SmAN11* | F | TCGGTATCTGCTGATGGGTC | [43] |
|  |  | R | TGTATCTCAAATCTTGCTTGTTCC |  |
| PGK | Control | F | TCGCTCTTGGAGAAGGTTGAC |  |
|  |  | R | CTTGTCGGCAATCACTACATCAG |  |
