# Supplementary figures and images for: Chalcone synthase (CHS) family members analysis from eggplant (Solanum melongena L.) in the flavonoid biosynthetic pathway and expression patterns in response to heat stress
Source: PLoS One. 2020 Apr 17;15(4):e0226537. doi: 10.1371/journal.pone.0226537 (PMC7164647; doi:10.1371/journal.pone.0226537)

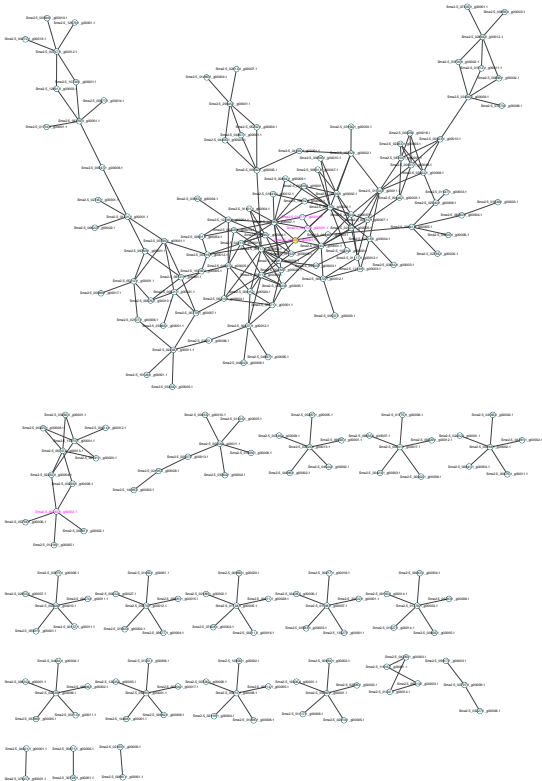

Supplement: S2 Fig — The pink labels represent the CHS gene family. (PDF) [file pone.0226537.s005.pdf]
