# Supplementary material for: Chalcone synthase (CHS) family members analysis from eggplant (Solanum melongena L.) in the flavonoid biosynthetic pathway and expression patterns in response to heat stress
Source: PLoS One. 2020 Apr 17;15(4):e0226537. doi: 10.1371/journal.pone.0226537 (PMC7164647; doi:10.1371/journal.pone.0226537)
Supplement: Attachment — Submitted filename: PONE-D-19-33168_R1.pdf [file pone.0226537.s007.pdf]

PLOS ONE

Chalcone synthase (CHS) family members analysis from eggplant (Solanum melongena L.) in the flavonoid biosynthetic pathway and expression patterns in response to heat stress

--Manuscript Draft--

**Manuscript Number:**	PONE-D-19-33168R1
**Article Type:**	Research Article
**Full Title:**	Chalcone synthase (CHS) family members analysis from eggplant (Solanum melongena L.) in the flavonoid biosynthetic pathway and expression patterns in response to heat stress
**Short Title:**	CHS family analysis from eggplant in flavonoid biosynthetic pathway under heatstress
**Corresponding Author:**	Xuexia Wu, Ph.D.<br>Shanghai Academy of Agriculture Sciences<br>Shanghai, CHINA
**Keywords:**	CHS family members; eggplant; anthocyanin biosynthesis; heat stress
**Abstract:**	Enzymes of the chalcone synthase (CHS) family participate in the synthesis of multiple secondary metabolites in plants, fungi and bacteria. CHS showed a significant correlation with the accumulation patterns of anthocyanin. The peel color, which isprimarily determined by the content of anthocyanin, is an economically important trait for eggplants that is affected by heat stress. A total of 7 CHS (SmCHS1-7) putative genes were identified in a genome-wide analysis of eggplants (S. melongena L.). The SmCHS genes were distributed on 7 scaffolds and were classified into 3 clusters. Phylogenetic relationship analysis showed that 73 CHS genes from 7 Solanaceae species were classified into 10 groups. SmCHS5 , SmCHS6 and SmCHS7 were continuously down-regulated under 38 ℃ and 45 ℃ treatment, while SmCHS4 was up-regulated under 38 ℃ but showed little change at 45 ℃ in peel. Expression profiles of key anthocyanin biosynthesis gene families showed that the PAL, 4CL and AN11 genes were primarily expressed in all five tissues. The CHI, F3H, F3'5'H, DFR, 3GT and bHLH1 genes were expressed in flower and peel. Under heat stress, the expression level of 52 key genes were reduced under heat stress. In contrast, the expression patterns of eight key genes similar to SmCHS4 were up-regulated at 38 ℃ 3h. Comparative analysis of putative CHS protein biochemical characteristics, cis - regulatory elements, and regulatory networks indicated that SmCHS gene family has a conserved gene structure and functional diversification. SmCHS showed two or more expression patterns and performed multiple functions to regulate anthocyanin content. Combined with analysis of regulatory networks, to the results of this study may facilitate further research to understand the regulatory mechanism governing peel color in eggplants.
**Order of Authors:**	Xuexia Wu, Ph.D.
	Shengmei Zhang
	Xiaohui Liu
	Jing Shang
	Aidong Zhang
	Zongwen Zhu
	Dingshi Zha
**Response to Reviewers:**	Dear Reviewers<br>Thank you and anonymous reviewers very much for your kind and useful comments for our manuscript "Genome-wide analysis of chalcone synthase (CHS) family from eggplant (Solanum melongena L.) in flavonoid biosynthetic pathway and expression pattern in response to heat stress" (PONE-D-19-33168). We have read and seriously considered the comments very carefully, checked and revised the manuscript many times. Major revisions throughout the revised manuscript have been made as followings.

Powered by Editorial Manager® and ProduXion Manager® from Aries Systems Corporation

1. The title name have been changed to “Chalcone synthase (CHS) family analysis from eggplant (Solanum melongena L.) in flavonoid biosynthetic pathway and expression pattern in response to heat stress”
2. The ‘Introduction’ section has been modified according to the comments
3. The results of all 73 CHS proteins sequence alignment, conserved residues and sequence diversity were analyzed.
4. We corrected some other grammatical errors and words spelling mistakes.

The followings are responses to the comments point-by-point.
Review Comments to the Author
Reviewer #1: In this manuscript entitled ‘Genome-wide analysis of chalcone synthase (CHS) family from eggplant (Solanum melongena L.) in flavonoid biosynthetic pathway and expression pattern in response to heat stress’ the authors have identified CHS-encoding genes of eggplant, performed their in silico characterization and analysed the expression pattern of key genes associated with anthocyanin biosynthesis pathways. Here are the following comments on the research for the authors:
1. The manuscript need to be revised for its English, grammar and flow of information. For example in L130: ‘The profiles of CHS (PF00195 and PF02797) were downloaded from the Pfam’- wwere need to be corrected to were. Line 198: ‘the other 7 Solanaceae species ranged from 17.3 to 47.4, the length of protein ranged’ kDa need to be added to the molecular weights mentioned here.
Answer: Thank you for pointing out the mistakes in the manuscript. We have corrected these issues in the revised manuscript according to comments, and checked the manuscript many times. Please refer to line 163 in revised manuscript with track changes.

2. It would be more informative for the readers if authors will provide a comparative mapping of CHS genes in the genome of other related plants where sequence information are available.
Answer: Chromosomal location of CHS genes were added in Table S1. Sequence alignment of all 73 CHS proteins of Solanum specie showed in the Fig S1. The VIII, IX and X groups are distinguished from other groups mainly depends on the position 1-164 amino acids, GroupsI, II and III are relatively conservative at the position 260-360 amino acids, in which the other groups are very diverse(S1 Fig). Please refer to line 325-328.

3. Fig. 7. represents key anthocyanin biosynthesis gene expression profiles in response to heat stress. A bar diagram with proper error bars for individual gene will be the most appropriate way for presentation of this result. The authors are advised to prepare the same for readers convenient.
Answer: Thanks for your suggestion. Error bars have been added in the Fig7.

4. Authors have performed the phylogenetic classification of CHS gene in Solanaceae and found that 73 CHS genes have classified into 10 groups. However, it has not been mentioned or discussed that on what basis the CHS are classified and how one group differs with the others.
Answer: The results of all 73 CHS proteins sequence alignment were added in S1 Fig. Conserved residues and sequence diversity of 73 CHS proteins were analyzed. Please refer to line 240-244 and 325-328

5. As authors have mentioned in the text about some of the cis regulatory element present on all the SmCHS promoter, similarly they should mention the unique cis element for each SmCHS genes. Not all CHS genes had shown similar expression pattern, thus a probable correlation of cis regulatory element on promoter and expression pattern of the gene should be mentioned in the discussion.
Answer: I'm sorry we miss the point. We have added this to the ‘result’ section refer to line 303-307.

Reviewer #2: The manuscript entitled “Genome-wide analysis of chalcone synthase (CHS) family from eggplant (Solanum melongena L.) in flavonoid biosynthetic pathway and expression pattern in response to heat stress” by Wu et al. reports the in silico analysis of chalcone synthase (CHS) gene family in eggplant. In brief, the current manuscript presents biochemical characteristics, phylogenetic relationships, gene

structures, cis-regulatory elements, regulatory network and functional predictions of the CHS gene family members.
The current quality of the manuscript leaves a number of questions to be answered, which are outlined below. The exposition of the results and its discussion in the manuscript needs a major revision including careful English usage and style edition.
1. The manuscript title starts with ‘Genome-wide analysis’; however, most of the analysis has been performed on CHS proteins. The work performed in the manuscript does not justify the title.
Answer: We changed the manuscript title as to ‘Chalcone synthase (CHS) family members analysis from eggplant (Solanum melongena L.) in the flavonoid biosynthetic pathway and expression patterns in response to heat stress’.

2. The ‘Introduction’ section of the manuscript is not coherent. Also, it does not describe the relevance of studying CHS gene family in proper manner.
Answer: We have added more information about CHS involve in synthesis of flavonoid compounds during heat stress defense. Please refer to line 70-80.

3. One of the major concerns is, authors have given RNA-Seq data in the result section. However, the source of this data is not described in the “Material and Method” section.
Answer: The overview of RNA-seq result from our lab was published (Zhang S et al, BMC Plant Biol. 2019 (1): 1-13). We therefore added a brief description of data source about RNA-seq in the“Material and Method” section. Please refer to line 196-198.

4. Also, authors have not provided the proper statistics of the validation experiment. Statistical analysis should be done to evaluate the significance of qRT-PCR data. It is recommended to include statistical analysis in the ‘Materials and methods’ section.
Answer: we have added the statistical analysis information of qRT-PCR in the ‘Materials and methods’ section. Please refer to line 223-225.

5. Writing in Subheadings 4 to 7 under the ‘Results’ section is very technical. The data is not described properly. Authors have chosen to explain the dataset in the ‘Discussion’ section. The outcome of the experiments should be described in the ‘Results’ section and interpretation in consistence with the previous studies should be given in the ‘Discussion’ section.
Answer: We agree and have revised the ‘results’ and ‘discussion’ accordingly. Please refer to line 351-361.

6. In Figure 3A, the abundance of cis-elements is given in all the CHS genes in eggplant. How the regulation of all the genes can be shown together?
Answer: We changed the histogram of figure 3A into a table to help readers understand the results.

7. In Figure 5, there is no downregulation value. How the data has been calculated? What control has been taken and how the data has been normalized?
Answer: Some errors have been corrected in line 351 of manuscript. Three SmCHS genes (SmCHS1, SmCHS2, and SmCHS3) were continuously downregulated under 38 ℃ and 45 ℃ treatment compared with the CK (27 ℃). Gene expression level was estimated by RNA-seq from mean FPKM (fragments per kilobase of exon model per million reads mapped) values for each treatment, and showed the expression patterns in heatmap. Each treatment had three biological replicates.

8. Figure 6, network is not very clear. A clear image has to be provided.
Answer: Is the network image you referred to Figure S2? We're sorry that there are too many genes in the network image. The picture can be expanded up to 6400%, so that the above words can be read clearly.

9. In ‘Material and Method’ section, what is CK? Why the samples harvested at 28º C are termed as CK and all the subsequent data is compared to CK?
Answer: The optimum growth temperature for eggplant is between 22 and 30°C. Eggplants subjected to high temperature may exhibit to stagnation of growth, abortion of flower buds, and decrease of pollen viability rate and fruit set, and the peel’s color will turn light when the temperature is over 35°C. Therefore, samples of 27 °C were termed as CK.

Powered by Editorial Manager® and ProduXion Manager® from Aries Systems Corporation

	10. In Figure 7, all the genes are showing high expression at 3 h CK. Do authors have any explanation of these results? Also, this data has been recorded from which tissue?<br>Answer: Under heat stress, SmCHS4 and some anthocyanin biosynthesis related genes show different expression profiles at 38 ℃-3h. These results suggest that these co-up-regulated genes contribute to protect the eggplant at beginning of heat stress defense. These data were recorded from the eggplant peels, which is indicated in the Figure 5 and Figure 6.<br><br>11. The other major issue is that this manuscript requires a thorough language editing since there are numerous grammatical errors including dropped articles, split infinitives, improper word usage etc. It is advisable that the manuscript must be edited by an English-speaking personal.<br>Answer: We checked and revised the manuscript many times. Some grammatical errors and words spelling mistakes have been modified by English editing company of American Journal Experts (AJE).<br><br>12. Reference section has to be rechecked. For example, in few places journal name is abbreviated (J Exp Bot) and in few places it is not (Plant physiology). In few references, journal name is capitalized and in others it is not.<br>Answer: This issue has been corrected.<br><br>13. Biochemical analysis of CHS proteins (pI, molecular weight etc.) is not relevant to the manuscript. This data can be removed from the draft.<br>Overall, the manuscript is not of adequate quality. The manuscript should be revised thoroughly for data presentation, result interpretation, description and language.<br>Answer: Biochemical analysis of CHS proteins was removed.
**Additional Information:**	
**Question**	**Response**
**Financial Disclosure**<br><br>Enter a financial disclosure statement that describes the sources of funding for the work included in this submission. Review the submission guidelines for detailed requirements. View published research articles from *PLOS ONE* for specific examples.<br><br>This statement is required for submission and **will appear in the published article** if the submission is accepted. Please make sure it is accurate.	YES-Specify the role(s) played

**Unfunded studies**<br>Enter: *The author(s) received no specific funding for this work.*<br><br>**Funded studies**<br>Enter a statement with the following details:<br>• Initials of the authors who received each award<br>• Grant numbers awarded to each author<br>• The full name of each funder<br>• URL of each funder website<br>• Did the sponsors or funders play any role in the study design, data collection and analysis, decision to publish, or preparation of the manuscript?<br>• **NO** - Include this sentence at the end of your statement: *The funders had no role in study design, data collection and analysis, decision to publish, or preparation of the manuscript.*<br>• **YES** - Specify the role(s) played.<br><br>* typeset	
**Competing Interests**<br><br>Use the instructions below to enter a competing interest statement for this submission. On behalf of all authors, disclose any competing interests that could be perceived to bias this work—acknowledging all financial support and any other relevant financial or non-financial competing interests.<br><br>This statement **will appear in the published article** if the submission is accepted. Please make sure it is accurate. View published research articles from *PLOS ONE* for specific examples.	I have read the journal's policy and the authors of this manuscript have the following competing interests

Powered by Editorial Manager® and ProduXion Manager® from Aries Systems Corporation

**NO authors have competing interests**<br><br>Enter: *The authors have declared that no competing interests exist.*<br><br>**Authors with competing interests**<br><br>Enter competing interest details beginning with this statement:<br><br>*I have read the journal's policy and the authors of this manuscript have the following competing interests: [insert competing interests here]*<br><br>* typeset	
**Ethics Statement**<br><br>Enter an ethics statement for this submission. This statement is required if the study involved:<br><br>• Human participants<br>• Human specimens or tissue<br>• Vertebrate animals or cephalopods<br>• Vertebrate embryos or tissues<br>• Field research<br><br>Write "N/A" if the submission does not require an ethics statement.<br><br>General guidance is provided below. Consult the submission guidelines for detailed instructions. **Make sure that all information entered here is included in the Methods section of the manuscript.**	N/A

**Format for specific study types**<br><br>**Human Subject Research (involving human participants and/or tissue)**<br>• Give the name of the institutional review board or ethics committee that approved the study<br>• Include the approval number and/or a statement indicating approval of this research<br>• Indicate the form of consent obtained (written/oral) or the reason that consent was not obtained (e.g. the data were analyzed anonymously)<br><br>**Animal Research (involving vertebrate animals, embryos or tissues)**<br>• Provide the name of the Institutional Animal Care and Use Committee (IACUC) or other relevant ethics board that reviewed the study protocol, and indicate whether they approved this research or granted a formal waiver of ethical approval<br>• Include an approval number if one was obtained<br>• If the study involved *non-human primates*, add *additional details* about animal welfare and steps taken to ameliorate suffering<br>• If anesthesia, euthanasia, or any kind of animal sacrifice is part of the study, include briefly which substances and/or methods were applied<br><br>**Field Research**<br><br>Include the following details if this study involves the collection of plant, animal, or other materials from a natural setting:<br>• Field permit number<br>• Name of the institution or relevant body that granted permission	
**Data Availability**<br><br>Authors are required to make all data underlying the findings described fully available, without restriction, and from the time of publication. PLOS allows rare exceptions to address legal and ethical concerns. See the PLOS Data Policy and FAQ for detailed information.	Yes - all data are fully available without restriction

A Data Availability Statement describing where the data can be found is required at submission. Your answers to this question constitute the Data Availability Statement and **will be published in the article**, if accepted.<br><br>**Important:** Stating 'data available on request from the author' is not sufficient. If your data are only available upon request, select 'No' for the first question and explain your exceptional situation in the text box.<br><br>Do the authors confirm that all data underlying the findings described in their manuscript are fully available without restriction?	
**Describe where the data may be found in full sentences. If you are copying our sample text, replace any instances of XXX with the appropriate details.**<br><br>• If the data are **held or will be held in a public repository**, include URLs, accession numbers or DOIs. If this information will only be available after acceptance, indicate this by ticking the box below. For example: *All XXX files are available from the XXX database (accession number(s) XXX, XXX.)*.<br>• If the data are all contained **within the manuscript and/or Supporting Information files**, enter the following: *All relevant data are within the manuscript and its Supporting Information files.*<br>• If neither of these applies but you are able to provide **details of access elsewhere**, with or without limitations, please do so. For example:<br><br>*Data cannot be shared publicly because of [XXX]. Data are available from the XXX Institutional Data Access / Ethics Committee (contact via XXX) for researchers who meet the criteria for access to confidential data.*<br><br>*The data underlying the results presented in the study are available from (include the name of the third party*	All relevant data are within the manuscript and its Supporting Information files.

Powered by Editorial Manager® and ProduXion Manager® from Aries Systems Corporation

and contact information or URL).<br>• This text is appropriate if the data are owned by a third party and authors do not have permission to share the data.<br><br>* typeset	
Additional data availability information:	

Powered by Editorial Manager® and ProduXion Manager® from Aries Systems Corporation

Manuscript Click here to access/download;Manuscript;Manuscript.docx

Chalcone synthase (CHS) family members analysis from eggplant (*Solanum melongena* L.) in the flavonoid biosynthetic pathway and expression patterns in response to heat stress

Xuexia Wu¶, Shengmei Zhang¶, Xiaohui Liu, Jing Shang, Aidong Zhang, Zongwen Zhu, Dingshi Zha*

Horticultural Research Institute, Shanghai Academy of Agricultural Sciences, Shanghai Key Laboratory of Protected Horticultural Technology, Shanghai, 201403, China *

Corresponding author: Dingshi Zha

E-mail: dingshizha1966@126.com

¶ These authors contributed equally to this work.

Abstract

Enzymes of the chalcone synthase (CHS) family participate in the synthesis of multiple secondary metabolites in plants, fungi and bacteria. CHS showed a significant correlation with the accumulation patterns of anthocyanin. The peel color, which isprimarily determined by the content of anthocyanin, is an economically important trait for eggplants that is affected by heat stress. A total of 7 *CHS* (*SmCHS1-7*) putative genes were identified in a genome-wide analysis of eggplants (*S. melongena* L.). The *SmCHS* genes were distributed on 7 scaffolds and were classified into 3 clusters. Phylogenetic relationship analysis showed that 73 *CHS* genes from 7 Solanaceae species were classified into 10 groups. *SmCHS5*, *SmCHS6* and *SmCHS7* were continuously down-regulated under 38 °C and 45 °C treatment, while *SmCHS4* was up-regulated under 38 °C but showed little change at 45 °C in peel. Expression profiles of key anthocyanin biosynthesis gene families showed that the PAL, 4CL and AN11 genes were primarily expressed in all five tissues. The CHI, F3H, F3'5'H, DFR, 3GT and bHLH1 genes were expressed in flower and peel. Under heat stress, the expression level of 52 key genes were reduced under heat stress. In contrast, the expression patterns of eight key genes similar to *SmCHS4* were up-regulated at 38 °C 3h. Comparative analysis of putative CHS protein biochemical characteristics, *cis*-regulatory elements, and regulatory networks indicated that *SmCHS* gene family has a conserved gene structure and functional diversification. *SmCHS* showed two or more expression patterns and performed multiple functions to regulate anthocyanin content. Combined with analysis of regulatory networks, to the results of this study may facilitate further research to understand the regulatory mechanism governing peel color in eggplants.

Introduction

Eggplant (S. *melongena* L.) is one of the most important thermophilic vegetables produced in many tropical and temperate regions around the world. The optimum growth temperature for eggplant is between 22 and 30 °C. Eggplants subjected to high temperature may exhibit to stagnation of growth, abortion of flower buds, and decrease

of pollen viability rate and fruit set, and the peel's color will turn light when the temperature is over 35 °C. High temperature severely reduces the yield and affects the appearance quality of eggplant. However, the molecular mechanism governing high temperature stress in eggplants has not been thoroughly elucidated.

Anthocyanins are plant secondary metabolites and are among the most abundant natural pigments, that are responsible for the characteristic colors in flowers, fruits and vegetables plant tissues. The anthocyanin biosynthesis pathway has been studied in numerous plant species and most of the genes involved in this process have been identified. Moreover, anthocyanins play an important role in plant survival under stressful environmental conditions. High temperatures are known to reduce anthocyanin accumulation and have discoloration effects in many plant tissues, causing drastic effects in colored flowers [1, 2], and affecting the skin of such fruits as grape berries, apples and eggplant [3-7].

It is well known that CHS is the gatekeeper of the anthocyanin pathway [8]. Enzymes of chalcone synthase (CHS) are member of the plants-specific type III polyketide synthase (PKS) [9, 10], family and catalyze the first committed step of the branch of the phenylpropanoid pathway, which leads to the synthesis of flavonoids [11, 12]. Flavonoids are well known as a group of plant secondary metabolites that comprise several different classes of compounds, such as chalcones, flavones, flavonol isoflavones and anthocyanins. Flavonoids have a wide variety of biological functions in, for instance, flower pigmentation, protection against UV radiation, pathogen defense, auxin transport and pollen fertility [13-15]. CHS also showed a significant correlation with synthesis of flavonoid compounds during heat stress defense. In bread wheat, heat stress responsive element has been found in the promoter of *Chs-D1* gene [16]. High-temperature stress had a large impact on the expression of *CHS7, CHS8* in both seeds and pods of Soybean [17]. The transcript levels of *CHS* decreased in apple peel and rose flower after heat treatment [1, 4]. In cork oak, *CHS* gene expression exhibited an increase under 45 °C, but showed a decreased expression at 55 °C [18]. The

emergence of *CHSV* and *CHSVII* is important for the development of fungal heat stress tolerance and pathogenicity in pathogenic fungi. [19]. In addition, *CHS* (Sme2.5_00283.1_g00002.1) was up-regulated, and the other two *CHS* gene members were down-regulated under heat stress in peel of eggplant [7].

The product of the CHS reaction is a pivotal precursor for a large array of secondary metabolites derived from malonyl-CoA and p-coumaroyl-CoA. CHS exists as homodimeric iterative PKS (monomer size of 42-45 kDa) with two independent active sites that catalyze a series of decarboxylation, condensation, and cyclization reactions [10, 20]. Member of the CHS superfamily share high similarity in their amino acid sequence, which contains the structurally conserved catalytic center consisting of four residues, Cys-His-Asn-Phe, and most of the genes contain two exons and one intron [21]. However, the *CHS* gene family has not been characterized in eggplants to date.

In the current study， all *SmCHS* family members were identified in eggplant. A comprehensive analysis of members was performed, including gene structures, the biochemical characteristics of putative CHS protein, promoter *cis*-elements, phylogenetic relationships among members in other relative species, and their expression profiles in various organs/tissues under high temperature stress. The findings of the present study may facilitate functional studies on eggplant *SmCHS* family genes.

Materials and methods

Plant materials and RNA extraction

The eggplant cultivar ‘Tewangda’ is a cold-tolerant cultivar with blackish purple skin. This cultivar grows vigorously and has good fruit setting. The fruit shape has a 27.6 cm fruit length, a 5.4 cm transverse diameter and a 209 g single fruit weight on average. The ‘Tewangda’ fruit has good commercial properties and good transportation resistance. ‘Tewangda’ fruits were grown at the same growth stage and were randomly

selected. These plants were grown 144 days after sowing, and then placed inside incubators set at 27 ℃ (CK), 38 ℃ or 45 ℃ for 3 or 6 h (three plants per treatment). For each treatment, the tissue samples of root, stem, leaf, flower and peel were obtained and immediately frozen in liquid nitrogen and stored at -80 °C for RNA extraction and other analyses. All plant materials examined in this study were obtained from Shanghai Academy of Agricultural Sciences. Total RNA was extracted from each tissue sample using the mirVana miRNA Isolation Kit (Ambion) following the manufacturer's protocol. The extracted total RNA was stored at -80 °C. RNA integrity was evaluated using the Agilent 2100 Bioanalyzer (Agilent Technologies, Santa Clara, CA, USA).

Identification of the CHS family members in the eggplant genome

The whole protein sequence of *Solanum melongena* L. (eggplant) were obtained from the Eggplant Genome DataBase (http://eggplant.kazusa.or.jp) [22], and those of *Solanum tuberosum* L. (potato, http://solanaceae.plantbiology.msu.edu/pgsc_download.shtml) [23], *Solanum lycopersicum* (tomato, https://solgenomics.net/organism/Solanum_lycopersicum/genome) [24], *Solanum penellii* (wild tomato, https://www.plabipd.de/project_spenn/start.ep) [25], *Capsicum annuum* L. (pepper, http://peppergenome.snu.ac.kr) [26], *Petunia axillaris* (https://solgenomics.net/organism/Petunia_axillaris/genome) [27], *Petunia inflate* (https://solgenomics.net/organism/Petunia_inflata/genome) [27], and *Nicotiana tabacum* (common tobacco, https://www.ncbi.nlm.nih.gov/nuccore/AYMY00000000) [28]. The profiles of CHS (PF00195 and PF02797) were downloaded from the Pfam protein family database (http://pfam.xfam.org/), and these profile sequences were used as queries to perform BLASTP searches against the protein sequence data of all the species mentioned above with a maximum E-value of 1×10^{-3}, respectively [29]. To further verify the exact copy number of CHS and remove redundant sequences, the Pfam database and Genome websites were also searched using "chalcone synthase" as

keywords. All CHS sequences were submitted to EXPASy (https://web.expasy.org/protparam/) to calculate the number of amino acids, molecular weights and theoretical isoelectric points (pI).

Structural characterization

The locations and intron numbers of CHS were acquired through the genome website. All of the acquired protein sequences were first aligned by ClustalX software with the default parameters [30]. An unrooted maximum-likelihood phylogenetic tree was constructed using MEGA6 software with a bootstrap test of 1000 times [31]. The MEME program (Version 5.0.5, http://meme-suite.org/tools/meme) was used to identify the conserved motif of the CHS sequences with the following parameters: any number of repetitions, maximum of 10 misfits and optimum motif width of 6-200 amino acid residues. The WoLF PSORT program was used to predict the subcellular localization information of CHS proteins (https://www.genscript.com/wolf-psort.html) [32].

Analysis of *cis*–acting elements in SmCHS

The upstream sequences (2 kb) of the *SmCHS* coding sequences in eggplant were retrieved from the genome sequence and then submitted to PlantCARE (http://bioinformatics.psb.ugent.be/webtools/plantcare/html/) to identify regulatory elements [33].

Phylogenetic analysis of CHS genes

The full-length protein sequences of all eight species in Solanaceae were used for phylogenetic analysis. All of the protein sequences were first aligned by ClustalX software with the default parameters [30]. The phylogenetic tree was generated with MEGA6 software with a bootstrap test of 1000 times. The final tree was viewed and modified in Evolview software [34]. The CHS genes were classified into different groups according to the topology of the phylogenetic tree.

Expression analysis of antyocyanin biosynthetic genes and construction of the mRNA regulatory network

The RNA-seq results were obtained by our lab [35]. Gene expression level was estimated from mean FPKM (fragments per kilobase of exon model per million reads mapped) values for each treatment, and showed the expression patterns in heatmap.Significant differentially expressed genes (fold change ≥ 2 and *p*-value ≤ 0.05) were used to calculate the Pearson correlation coefficient between *CHS* genes and other genes. The TBtools program was used to elucidate the Gene Ontology (GO) functional classification for the mRNAs with correlation coefficients greater than 0.9 [36]. The top 5 regulatory mRNAs annotated by GO enrichment for the genes associated with anthocyanin biosynthesis were collected to construct the regulatory network. The network was visualized using Cytoscape [37].

qRT-PCR analysis

Expression level of anthocyanin biosynthesis genes, including phenylalanine ammonia lyase (PAL), cinnamate 4-hydroxylase (C4H), 4-coumarateCoA ligase (4CL), CHS, chalcone isomerase (CHI), flavanone 3-hydroxylase (F3H), flavonoid 3′-hydroxylase (F3′H), flavonoid 3′5′-hydroxylase (F3′5′H), dihydroflavonol4-reductase (DFR), anthocyanidin synthase (ANS), anthocyanidin 3-O-glucosyltransferase (3GT), Anthocyanins11 (AN11), and most transcription factors, such as myeloblastosis (MYB), basic helix-loop-helix (bHLH) and metadata authority description schema(MADS1), were analyzed. First-strand cDNA was synthesized from 1 μg from 5 tissues (root, stem, leaf, flower and peel) using a Prime Script RT Reagent Kit (Takara, Dalian, China). The qRT-PCR reactions were performed in 96-well plates using the ABI 7500 fast Real-Time PCR system (Applied Biosystems, USA) with the QuantiFast SYBR Green PCR Kit (Qiagen, Duesseldorf, Germany). The qRT-PCR parameters were as follows: 95 ℃ for 5 min, then 45 cycles of 95 ℃ for 10 s, 60 ℃ for 10 s, and 72 ℃ for 10 s. The relative mRNA expression levels were calculated using the $2^{-\Delta\Delta CT}$ method [38]. PGK(JX154676)

was used as an internal control to normalize the data. For each sample, three biological repeats were performed, the relative expression levels were calculated using the standard curve and normalized by the control's expression, the results were display by heatmap. The primer sequences are listed in Table S3.

Results

Identification of *CHS* genes and sequence analysis in Solanaceae species

A total of 7 *CHS* (*SmCHS1-7*) genes in eggplant were identified after being verified by protein sequence analysis and BlAST search using the eggplant genome annotation database (Additional file 1 Table S1a). The length of SmCHS protein ranged from 327 to 396 amino acids (Table 1, Table S2). The PKS type III active sites of the enzymes and Phe215 connected with CoA binding are conserved among all SmCHS (S1 Fig). In addition, 66 *CHS* genes were characterized from 7 other Solanaceae species. The subfamily numbers of *CHS* genes ranged from 6 (*Solanum penellii*) to 13 (*Petunia axillaris*) (Table 1, Additional file 1 Table S1b-h). The the length for the other 7 Solanaceae species proteins ranged from 156 to 431 amino acids (Additional file 2 Table S1a-g). The average number of amino acids was calculated and then employed as a data set for each species. The correlation coefficients among the above data were all greater than 0.99. This finding suggests that *CHS* genes are conserved in Solanaceae species.

Table 1. Features of *SmCHS* genes identified in eggplant.

Gene Name	Gene ID	Number of amino acids
SmCHS1	Sme2.5_01077.1_g00016.1	333
SmCHS2	Sme2.5_02154.1_g00001.1	389
SmCHS3	Sme2.5_13923.1_g00001.1	389
SmCHS4	Sme2.5_00283.1_g00002.1	392
SmCHS5	Sme2.5_01039.1_g00002.1	327
SmCHS6	Sme2.5_00346.1_g00019.1	396

SmCHS7	Sme2.5_05261.1_g00004.1	383

Structure and conserved motif analysis of *SmCHS*

The 7 *SmCHS* genes were distributed on 7 scaffolds. To better understand the evolution of *SmCHS* genes, an unrooted maximum-likelihood tree was constructed based on the 7 SmCHS protein sequences, and the *SmCHS* were classified into 3 clusters (i, ii and iii) (Fig 1). Among the *SmCHS* genes, only one *SmCHS7* had three exons, and the others had two exons (Fig 1) based on information available from the genome annotation. These results suggest the potential diversity of the biological functions of the *SmCHS* genes in eggplants.

Fig 1. Phylogenetic relationship and gene structure analysis of *SmCHS* genes.

The phylogenetic tree was on the left of the figure and showed that the *SmCHS* genes were classified into three clusters (i, ii and iii). The exon/intron organization of *SmCHS* is shown on the right of the figure. For *SmCHS* gene organization, yellow boxes represent exons, black lines represent introns, and green boxes indicate upstream/downstream regions. The lengths of the exons and introns are drawn to scale.

To understand the functional diversification of *SmCHS*, the conserved motifs of these 7 protein sequences were identified by the MEME program, and 10 conserved motifs were detected in eggplant (Fig 2, Table 2). The Chal_sti_synt_C domain and Chal_sti_synt_N domain were included in motifs 1 and motifs 2, respectively. For all 7 eggplant SmCHS proteins, Motif 1 and motif 2 exist in all of them, motif 3 is only absent in *SmCHS5*, and motif 4 and motif 5 are only absent in *SmCHS1*. The N-terminal domain (PF00195) of the CHS protein contained motif 1 and the combination of motifs 3, 4, 6, 7 and 9. The C-terminal domain (PF02797) of the CHS protein contained motif 2 and the combination of motifs 5, 8 and 10. Therefore, the motif configuration of the SmCHS reflects the conservation and diversity of the CHS family. To further

investigate the subcellular localization information of SmCHS proteins, the WoLF PSORT program was used to predict the localization of SmCHS protein [31]. SmCHS7 was predicted to localize in the nucleus, and SmCHS4 and SmCHS6 were predicted to localize in the chloroplast. The others SmCHS proteins were predicted to localize in the cytoplasmic. The different compositions of the domains and subcellular localization may indicate functional diversity.

Table 2 List of the putative motifs of CHS proteins

Motif	Width	Best possible match
Motif 1	167	IKEWGQPKSKITHLVFCTTSGVDMPGADYQLTKLLGLRPSVKRFMMYQQGCFAGGTVLRLAKDLAENNKGARVLVVCSEITAVGFRGPSETHPDSLVGQA
Motif 2	57	DWNSJFWIAHPGGPAILDQVELKLGLKPEKLRATRQVLSDYGNMSSACVLFILDEMR
Motif 3	56	RLCDKSMIKKRYMHLTEEILKENPNLCEYMAPSLDARQDIVVVEVPKLGKEAAQKA
Motif 4	38	QRAEGPATILAIGTATPSNCVDQSTYPDYYFRITNSEH
Motif 5	27	TTGEGLDWGVLLGFGPGLTIETIVLHS
Motif 6	11	LIEAFEPLGIS
Motif 7	8	MVTVEEVR
Motif 8	6	FCEKLI
Motif 9	7	QNIGKVN
Motif 10	7	ELKEKFK

Fig 2. Motifs conserved across all CHS proteins in eggplant. Ten conserved motifs are indicated in differently colored boxes.

Stress-related *cis*-elements in *SmCHS* promoters

To further study the potential regulatory mechanisms of *SmCHS* during abiotic stress responses, the 2 kb upstream sequences from the translation start sites of *SmCHS* were used to identify the *cis*-elements (Fig 3B). The results showed that all *SmCHS* had common upstream promoter elements, including TATA-box and CAAT-box, which occurred more than 100 times; therefore, these sequences were presumed to be the promoter sequences (Fig 3A). The elicitor response (ERE) and myeloblastosis (MYB)

occurred more than 10 times in the *SmCHS* upstream sequences. Research has shown that an increase in CHS activity causes a high accumulation of flavonoids that inhibits polar auxin transport [8, 39, 40]. Two *cis*-acting elements (ABRE, involved in abscisic acid responsiveness; AuxRR, involved in auxin responsiveness) were found in the upstream regions. MYB and myelocytomatosis (MYC) binding sites have also been identified, which may greatly influence plant stress tolerance. Cluster analysis of *cis*-element number showed that 7 *SmCHS* genes were divided into 3 groups (Ⅰ, Ⅱ, Ⅲ), and *SmCHS1*, *SmCHS2* and *SmCHS3* had similar regulatory pattern (Fig 3A). Five *cis*-elements (CARE, GCN4-motif, GT1-motif, MRE and TCT-motif) exist only in group Ⅰ, GARE-motif only exist in group Ⅲ. STRE exist in group Ⅱ and Ⅲ. These results showed that *SmCHS* is activated by a wide range of environmental and developmental stimuli, and there are many complex means of regulating *SmCHS* activity in eggplants.

Fig 3. *Cis*-elements in *CHS* family gene promoters. (A) Frequency of cis-element occurrence in upstream sequences. (B) Predicted *cis*-elements in *CHS* gene promoters. The scale bar indicates the length of promoters.

Phylogenetic analysis of *CHS* genes in Solanaceae

To analyze the evolutionary relationships of *CHS* genes in Solanaceae, an unrooted phylogenetic tree was constructed using full-length amino acid sequences. All 73 *CHS* genes were classified into 10 groups (Fig 4, Table 3), and the number of CHS gene groups ranged from two to eleven. The 7 SmCHS were categorized into 6 groups (groups Ⅰ, Ⅱ, Ⅶ, Ⅷ, Ⅸand Ⅹ), and group Ⅱ contained *SmCHS1* and *SmCHS2*. Groups Ⅰ, Ⅱ, Ⅸand Ⅹexist in all eight species, and groups Ⅲ, Ⅳand Ⅴwere absent in *Solanum melongena* L., *Solanum penellii*, *Solanum lycopersicum* and *Solanum tuberosum* L.. The group Ⅵis absent in *Capsicum annuum* L., *Nicotiana tabacum*, *Petunia inflate* and *Petunia axillaries* (Table 3). The Ⅷ, Ⅸ and Ⅹ groups are distinguished from other groups mainly depends on the position 1-164 amino acids, Groups Ⅰ, Ⅱ and Ⅲ are

relatively conservative at the position 260-360 amino acids, in which the other groups are very diverse(S1 Fig). These results suggested that the CHS were conserved, but small variations existed among the eight species in Solanaceae and showed that SmCHS1, SmCHS2 and SmCHS3 were more conserved than *SmCHS4* according to the phylogenetic tree.

Table 3. Distribution of *CHS* genes in the phylogenetic tree.

Plant Pecies	Number	Phylogenetic Group									
		I	II	III	IV	V	VI	VII	VIII	IX	X
Solanum melongena L.	7	1	2	0	0	0	0	1	1	1	1
Solanum penellii	6	1	1	0	0	0	0	1	1	1	1
Solanum lycopersicum	7	1	1	0	0	0	1	1	1	1	1
Solanum tuberosum L.	10	2	1	0	0	0	1	3	1	1	1
Capsicum annuum L.	9	1	1	0	2	0	0	2	1	1	1
Nicotiana tabacum	12	2	2	0	0	1	0	3	0	2	2
Petunia inflate	9	2	1	1	1	1	0	0	1	1	1
Petunia axillaris	13	1	1	3	1	3	0	0	1	1	2

Fig 4. Phylogenetic tree of *CHS* genes in Solanaceae species. The color region is associated with 10 groups of proteins (Group Ⅰ to Ⅹ).

Expression profile of key anthocyanin biosynthesis genes in eggplants under heat stress

Using the RNA-seq data, a heatmap of 96 key anthocyanin biosynthesis genes (PAL, C4H, 4CL, CHS, CHI, F3H/F3'H, F3'5'H, DFR, ANS, 3GT, MYB1, MYB2, bHLH1, AN11, MADS1) was established under heat stress (Fig 5). The expression of anthocyanidin synthase (ANS) and MYB2 was not identified during this sampling period. For seven *SmCHS* genes, three (*SmCHS5*, *SmCHS6*, and *SmCHS7*) were not identified, and the other four *SmCHS* genes were divided into two groups according to their expression patterns. Three of those four *SmCHS* genes (*SmCHS1*, *SmCHS2*, and *SmCHS3*) were continuously down-regulated under 38 ℃ and 45 ℃ treatment compared with the CK. However, *SmCHS4* was up-regulated under 38 ℃, but showed little change

at 45 ℃ in peel. These phenomena have also been observed in some other key gene families associated with anthocyanin biosynthesis. According to the RNA-seq results of 96 anthocyanin biosynthesis key genes in eggplant peel, *SmCHS4* showed the highest expression level at the 38 ℃-3h along with eight other genes (*Sme2.5_03336.1_g00008.1_PAL*, *Sme2.5_00041.1_g00017.1_4CL*, *Sme2.5_00283.1_g00002.1_smCHS4*, *Sme2.5_00298.1_g00002.1_F3H*, *Sme2.5_02066.1_g00012.1_F3H*, *Sme2.5_04260.1_g00001.1_F3H*, *Sme2.5_15970.1_g00001.1_F3H*, *Sme2.5_00670.1_g00012.1_DFR*, *Sme2.5_00747.1_g00013.1_AN11*) (Fig 6). In particular, *Sme2.5_03336.1_g00008.1_PAL* expression level under 38 ℃ doubled but was down-regulated at 45 ℃ compared with CK; *Sme2.5_00670.1_g00012.1_DFR*, *Sme2.5_00747.1_g00013.1_AN11* expression level increased 3-4 fold and 7-10 fold under 38 ℃, respectively.

Fig 5. Heatmap of 96 key anthocyanin biosynthesis genes expression level in eggplants peel under heat stress. The color box from blue to red indicate an increased expression level.

Fig 6. Expression profiles of *SmCHS4* and eight anthocyanin biosynthesis genes in response to heat stress. These genes have the highest expression level at 38 ℃-3h in eggplant peel. The error bars represent the standard error of the means of three biological replicates.

mRNA regulatory network associated with anthocyanin biosynthesis in eggplant

A total of 4928 mRNA correlation coefficients were more than 0.9, and all of these mRNAs were functionally categorized in the GO database. The top 20 GO enrichment results of biological processes are shown in Table 4. The function was involved in the regulation of biological processes (GO:0050789), regulation of cellular metabolic

processes (GO:0031323) and regulation of gene expression (GO:0010468) were collected and filtered to construct a regulatory network. In totally, 67 anthocyanin biosynthesis key genes and 146 regulatory mRNAs were included in this regulatory network (S2 Fig). These GO enrichment results suggest that the anthocyanin biosynthesis pathway may be regulated by a wide range of environmental and developmental stimuli.

Table 4. Top 20 GO enrichment results of biological processes.

GO term	GO ID	P value
cellular biosynthetic process	GO:0044249	0
cellular nitrogen compound biosynthetic process	GO:0044271	0
cellular response to chemical stimulus	GO:0070887	0
cellular response to stress	GO:0033554	0
regulation of biological process	GO:0050789	0
regulation of cellular macromolecule biosynthetic process	GO:2000112	0
developmental process	GO:0032502	0
regulation of RNA biosynthetic process	GO:2001141	0
regulation of cellular metabolic process	GO:0031323	0
cellular component organization	GO:0016043	0
response to organic substance	GO:0010033	1.11E-16
protein metabolic process	GO:0019538	1.11E-16
regulation of nitrogen compound metabolic process	GO:0051171	1.11E-16
regulation of gene expression	GO:0010468	1.11E-16
response to stimulus	GO:0050896	1.11E-16
regulation of nucleobase-containing compound metabolic process	GO:0019219	1.11E-16
response to chemical	GO:0042221	1.11E-16
cell communication	GO:0007154	1.11E-16
response to stress	GO:0006950	1.11E-16
oxoacid metabolic process	GO:0043436	2.22E-16

Expression pattern of anthocyanin biosynthesis key genes in different tissues under heat stress

Using the qRT-PCR data, a heatmap of 20 key anthocyanin biosynthesis genes was established in different tissues under heat stress (Fig 7). The qRT-PCR results showed a high consistency with the RNA-seq data, which suggested that the RNA-seq data were

credible. Most of the CHS genes were expressed in peel and were expressed at low levels in other tissues. The PAL, 4CL and AN11 genes were mainly expressed in all five tissues. The CHI, F3H, F3’5’H, DFR, 3GT and bHLH1 genes were expressed in flower and peel. MADS1 was expressed in stems, leaves, flowers and peels. Under heat stress， cluster i (cluster show in Fig 2) was continuously downregulation , cluster ii was up-regulated 4 times under 38 ℃compared with CK in peel, and cluster iii was not detected in most eggplant tissues.

Fig 7. Expression profiles of 20 key anthocyanin biosynthesis genes in different tissues.

Discussion

It is well-known that the *CHS* gene family plays a significant role in the growth and development of plants. In many species, multigene families of *CHS* have been identified. For example, six *CHS* genes have been described in turnip [41]. In maize, 14 complete *CHS* genes have been identified [42]. A total of 27 *CHS* genes were found in rice [43]. These studies showed that *CHS* members were divided into two or more subclasses according to phylogenetic analysis. Generally, genes grouped into the same subclasses shared similar evolutionary features, and obtained the same expression pattern. In our study, the identified sequences showed a high level of coding sequence similarity (above 90%). The *SmCHS* were classified into three clusters based on the results of the maximum-likelihood tree. At 35 ℃, previous studies showed that *SmCHS1* and *SmCHS3* (Sme2.5_01077.1_g00016.1, Sme2.5_13923.1_g00001.1) were down-regulated in peels of eggplant [7], which is in keeping with our results, other two clusters *CHS* genes show different expression patterns. These results suggest the functional diversification of *SmCHS*.

Flavonoids have numerous functions and contribute to pigments, signaling molecules, and protectants against biotic and abiotic stresses. The flavonoid biosynthetic pathway

is one of the most intensively investigated pathways for applied biological and genetic processes, as well as for understanding gene regulation, characterizing transposable elements and producing of agronomically stress-tolerant plants and natural dietary antioxidants. biosynthesis of anthocyanins responds to environmental stressors, such as light, nutrient depletion, and temperature change. The peel color determined by the content of anthocyanin is a majority economically important trait for eggplant, and this color is modulated by the genes in the flavonoid biosynthesis pathway. Compared with other tissues, *SmMYB1* and all anthocyanin biosynthetic key genes (*SmCHS*, *SmCHI*, *SmF3H*, *SmDFR*) except *SmPAL* were dramatically up-regulated in the fruit skin of the purple cultivar [44]. The full length cDNA of *SmCHS*, *SmCHI*, *SmF3'5'H*, and *SmDFR* were isolated from eggplants by Jiang. These genes have the highest expression levels in peels except for *SmF3H*, which was detected in stems [45]. The expression profiles of these key gene families under heat stress were investigated in our study. ‘These anthocyanin biosynthesis key genes (PAL, 4CL, AN11, CHI, F3H, F3’5’H, DFR, 3GT and bHLH1) show tissue specific expression, suggesting that these genes respond at the late stage of the anthocyanin pathway and directly regulate the color of fruit skin and flower.

Heat stress reduced the anthocyanin content and the enzyme activities of CHS, DFR, ANS and 3GT/UFGT in eggplant peel and strengthened the activity of PAL [35, 46]. When the temperature exceeds 35 ℃, the eggplant will be dehydrated and shrink, and the peel’s color will lighten. CHS is a key enzyme of the flavonoid biosynthesis pathway. Most of the genes associated with flavonoid biosynthesis were down-regulated under heat stress. In this study, the genes of the flavonoid biosynthesis pathway showed tissue-specificity, and genes expressed in different phases and tended to change over time (Fig 7). Under heat stress, *SmCHS4* and some anthocyanin biosynthesis related genes show different expression profiles at 38 ℃-3h (Fig 6), suggest that these co-up-regulated genes contribute to protect the eggplant at beginning of heat stress defense. In addition, 52 gene expression levels were reduced under heat stress, which was similar to Lv’s results [7], while 35 gene expression levels were not identified. These results suggest

that some key anthocyanin biosynthesis genes help to protect the eggplant from damage to heat stress. Moreover, these gene families exhibited two or more expression patterns and performed multiple genetic functions to regulate anthocyanin content. Combined with regulatory networks, it is possible to further understand the regulatory mechanism of peel color in eggplants.

Conclusions

In this study, a genome-wide analysis of the *SmCHS* gene family in eggplants was performed. The CHS protein biochemical characteristics, phylogenetic relationships, gene structures, *cis*-regulatory elements, regulatory network and functional predictions of the *smCHS* gene family members were examined. The *SmCHS* gene family has conserved gene structure and functional diversification. CHS plays important roles in the anthocyanin biosynthesis pathway, exhibits two or more expression patterns and executes multiple functions to regulate anthocyanin content in eggplant peels under heat stress. The result of this study may contribute to the production of eggplant for further research on the functions, regulation and evolution of the CHS family.

Author contributions

DZ proposed the research, and ZZ and AD carried out the preparation and treatment of test materials. XL and SJ designed and performed the experiment. XW and SZ analyzed the data and wrote the manuscript. XW revised the article. All authors read and approved the final manuscript.

Supporting Information

Table S1. CHS protein sequences of Solanum species.

Table S2. Features of CHS genes identified in Solanum species.

Table S3. Primers used for real time PCR analysis.

Fig S1. Sequence alignment of all 73 CHS proteins of Solanum specie. Color bars on the left represent the 10 groups in Fig 4. Active site residues are highlighted in yellow,

malony-CoA binding sites are highlighted in blue and other conserved sequence are shown in green.

Fig S2. Interaction network key to anthocyanin biosynthesis in eggplant. The pink labels represent the CHS gene family.

References:

1. Dela G, Or E, Ovadia R, Nissim-Levi A, Weiss D, Oren-Shamir M. Changes in anthocyanin concentration and composition in 'Jaguar' rose flowers due to transient high-temperature conditions. Plant Sci. 2003; 164(3): 333-340. https://doi.org/10.1016/S0168-9452(02)00417-X.

2. Nozaki K, Takamura T, Fukai S. Effects of high temperature on flower colour and anthocyanin content in pink flower genotypes of greenhouse chrysanthemum (*Chrysanthemum morifolium* Ramat.). J Hortic Sci Biotechnol. 2006; 81(4): 728-734. https://doi.org/10.1080/14620316.2006.11512130.

3. Mori K, Goto-Yamamoto N, Kitayama M, Hashizume K. Loss of anthocyanins in red-wine grape under high temperature. J Exp Bot. 2007; 58(8): 1935-1945. https://doi.org/10.1093/jxb/erm055 PMID: 17452755.

4. Lin-Wang K, Micheletti D, Palmer J, Volz R, Lozano L, Espley R, et al. High temperature reduces apple fruit colour via modulation of the anthocyanin regulatory complex. Plant Cell Environ. 2011; 34(7): 1176-1190. https://doi.org/10.1111/j.1365-3040.2011.02316.x PMID: 21410713.

5. Movahed N, Pastore C, Cellini A, Allegro G, Valentini G, Zenoni S, et al. The grapevine VviPrx31 peroxidase as a candidate gene involved in anthocyanin degradation in ripening berries under high temperature. J Plant Res. 2016; 129(3): 513-526. https://doi.org/10.1007/s10265-016-0786-3 PMID: 26825649.

6. Pastore C, Dal Santo S, Zenoni S, Movahed N, Allegro G, Valentini G, et al. Whole plant temperature manipulation affects flavonoid metabolism and the transcriptome of grapevine berries. Front Plant Sci. 2017; 8: 929. https://doi.org/10.3389/fpls.2017.00929 PMID: 28634482.

7. Lv LL, Feng XF, Li W, Li K. High temperature reduces peel color in eggplant (*Solanum melongena*) as revealed by RNA-seq analysis. Genome. 2019; 62(7): 503-512. https://doi.org/10.1139/gen-2019-0021 PMID: 31132325.

8. Dao T, Linthorst H, Verpoorte R. Chalcone synthase and its functions in plant resistance. Phytochem Rev. 2011; 10(3): 397. https://doi.org/DOI 10.1007/s11101-011-9211-7 PMID: 21909286.

9. Schröder J. A family of plant-specific polyketide synthases: facts and predictions. Trends Plant Sci. 1997; 2(10): 373-378. https://doi.org/10.1016/S1360-1385(97)01104-7.

10. Austin MB, Noel JP. The chalcone synthase superfamily of type III polyketide synthases. Nat Prod Rep. 2003; 20(1): 79-110. https://doi.org/10.1039/b100917f PMID: 12636085.

11. Winkel-Shirley B. It takes a garden. How work on diverse plant species has contributed to an understanding of flavonoid metabolism. Plant Physiol. 2001; 127(4): 1399-1404. https://doi.org/10.1104/pp.127.4.1399 PMID: 11743081.

12. Pang Y, Shen G, Wu W, Liu X, Lin J, Tan F, et al. Characterization and expression of chalcone synthase gene from *Ginkgo biloba*. Plant Sci. 2005; 168(6): 1525-1531. https://doi.org/10.1016/j.plantsci.2005.02.003 PMID: 24841790.

13. Winkel-Shirley B. Biosynthesis of flavonoids and effects of stress. Curr Opin plant Bio. 2002; 5(3): 218-223. https://doi.org/10.1016/S1369-5266(02)00256-X PMID: 11960739.

14. Buer CS, Imin N, Djordjevic MA. Flavonoids: new roles for old molecules. J Integr plant biol. 2010; 52(1): 98-111. https://doi.org/CNKI:SUN:ZWXB.0.2010-01-012 PMID: 20074144.

15. Falcone FM, Rius SP, Casati P. Flavonoids: biosynthesis, biological functions, and biotechnological applications. Front Plant Sci. 2012; 3: 222. https://doi.org/10.3389/fpls.2012.00222 PMID: 23060891.

16. Glagoleva AY, Ivanisenko NV, Khlestkina EK. Organization and evolution of the chalcone synthase gene family in bread wheat and relative species. BMC Genet. 2019; 20(1): 30. https://doi.org/10.1186/s12863-019-0727-y PMID: 30885129.

17. Chennupati P, Seguin P, Chamoun R, Jabaji S. Effects of high-temperature stress on soybean isoflavone concentration and expression of key genes involved in isoflavone synthesis. J AgricFood Chem. 2012; 60(51): 12421-12427. https://doi.org/10.1021/jf3036319 PMID: 23199070.

18. Correia B, Rodriguez JL, Valledor L, Almeida T, Santos C, Cañal MJ, et al. Analysis of the expression of putative heat-stress related genes in relation to thermotolerance of cork oak. J Plant Physiol. 2014; 171(6): 399-406. https://doi.org/10.1016/j.jplph.2013.12.004 PMID: 24594391.

19. Liu R, Xu C, Zhang Q, Wang S, Fang W. Evolution of the chitin synthase gene family correlates with fungal morphogenesis and adaption to ecological niches. Sci Rep. 2017; 7: 44527. https://doi.org/10.1038/srep44527 PMID: 28300148.

20. Tropf S, Kärcher B, Schröder G, Schröder J. Reaction Mechanisms of Homodimeric Plant Polyketide Synthases (Stilbene and Chalcone Synthase). A single active site for the condensing reaction is sufficient for synthesis of stilbenes, chalcones, and 6'-deoxychalcones. J Biol Chem. 1995; 270(14): 7922-7928. https://doi.org/10.1074/jbc.270.14.7922 PMID: 7713888.

21. Ferrer J-L, Jez JM, Bowman ME, Dixon RA, Noel JP. Structure of chalcone synthase and the molecular basis of plant polyketide biosynthesis. Nat Struct Mol Biol. 1999; 6(8): 775. https://doi.org/10.1038/11553 PMID: 10426957.

22. Hideki H, Kenta S, Koji M, Tsukasa N, Satomi N, Akio O, et al. Draft genome sequence of eggplant (*Solanum melongena* L.): the representative *Solanum* species indigenous to the old world. DNA Res. 2014; 21(6): 649-660. https://doi.org/10.1093/dnares/dsu027 PMID: 25233906.

23. Consortium PGS. Genome sequence and analysis of the tuber crop potato. Nature. 2011; 475(7355): 189. https://doi.org/10.1038/nature10158 PMID: 21743474.

24. Consortium TTG. The tomato genome sequence provides insights into fleshy fruit evolution. Nature. 2012; 485(7400): 635. https://doi.org/10.1038/nature11119.

25. Schmidt MH-W, Vogel A, Denton AK, Istace B, Wormit A, van de Geest H, et al. De novo assembly of a new *Solanum pennellii* accession using nanopore sequencing. Plant Cell. 2017; 29(10): 2336-2348. https://doi.org/10.1105/tpc.17.00521 PMID:

29025960.
26. Kim S, Park M, Yeom S-I, Kim Y-M, Lee JM, Lee H-A, et al. Genome sequence of the hot pepper provides insights into the evolution of pungency in *Capsicum* species. Nat Genet. 2014; 46(3): 270. https://doi.org/10.1038/ng.2877 PMID: 24441736.
27. Bombarely A, Moser M, Amrad A, Bao M, Bapaume L, Barry CS, et al. Insight into the evolution of the Solanaceae from the parental genomes of *Petunia hybrida*. Nat Plants. 2016; 2(6): 16074. https://doi.org/10.1038/nplants.2016.74 PMID: 27255838.
28. Sierro N, Battey JN, Ouadi S, Bakaher N, Bovet L, Willig A, et al. The tobacco genome sequence and its comparison with those of tomato and potato. Nat Commun. 2014; 5: 3833. https://doi.org/10.1038/ncomms4833 PMID: 24807620
29. Boratyn GM, Camacho C, Cooper PS, Coulouris G, Fong A, Ma N, et al. BLAST: a more efficient report with usability improvements. Nucleic Acids Res. 2013; 41(W1): W29-W33. https://doi.org/10.1093/nar/gkt282 PMID: 23609542.
30. Thompson JD, Gibson TJ, Plewniak F, Jeanmougin F, Higgins DG. The CLUSTAL_X windows interface: flexible strategies for multiple sequence alignment aided by quality analysis tools. Nucleic Acids Res. 1997; 25(24): 4876-4882. https://doi.org/10.1093/nar/25.24.4876 PMID: 9396791.
31. Tamura K, Stecher G, Peterson D, Filipski A, Kumar S. MEGA6: molecular evolutionary genetics analysis version 6.0. Mol Biol Evol. 2013; 30(12): 2725-2729. https://doi.org/10.1093/molbev/mst197 PMID: 24132122
32. Horton P, Park K-J, Obayashi T, Fujita N, Harada H, Adams-Collier C, et al. WoLF PSORT: protein localization predictor. Nucleic Acids Res. 2007; 35(suppl_2): W585-W587. https://doi.org/10.1093/nar/gkm259 PMID: 17517783.
33. Lescot M, Déhais P, Thijs G, Marchal K, Moreau Y, Van de Peer Y, et al. PlantCARE, a database of plant *cis*-acting regulatory elements and a portal to tools for *in silico* analysis of promoter sequences. Nucleic Acids Res. 2002; 30(1): 325-327. https://doi.org/10.1093/nar/30.1.325 PMID: 11752327.
34. Subramanian B, Gao S, Lercher MJ, Hu S, Chen W-H. Evolview v3: a webserver for visualization, annotation, and management of phylogenetic trees. Nucleic Acids Res. 2019. https://doi.org/10.1093/nar/gkz357 PMID: 31114888.

35. Zhang S, Zhang A, Wu X, Zhu Z, Yang Z, Zhu Y, et al. Transcriptome analysis revealed expression of genes related to anthocyanin biosynthesis in eggplant (*Solanum melongena* L.) under high-temperature stress. BMC Plant Biol. 2019; 19(1): 1-13. https://doi.org/10.1186/s12870-019-1960-2 PMID: 31492114.

36. Chen C, Xia R, Chen H, He Y. TBtools, a Toolkit for Biologists integrating various biological data handling tools with a user-friendly interface. BioRxiv. 2018: 289660. https://doi.org/10.1101/289660.

37. Smoot ME, Ono K, Ruscheinski J, Wang P-L, Ideker T. Cytoscape 2.8: new features for data integration and network visualization. Bioinformatics. 2010; 27(3): 431-432. https://doi.org/10.1093/bioinformatics/btq675 PMID: 21149340.

38. Livak KJ, Schmittgen TD. Analysis of relative gene expression data using real-time quantitative PCR and the $2^{-\Delta\Delta CT}$ method. Methods. 2001; 25(4): 402-408. https://doi.org/10.1006/meth.2001.1262 PMID: 11846609.

39. Jacobs M, Rubery PH. Naturally occurring auxin transport regulators. Science. 1988; 241(4863): 346-349. https://doi.org/10.1126/science.241.4863.346 PMID: 17734864.

40. Brown DE, Rashotte AM, Murphy AS, Normanly J, Tague BW, Peer WA, et al. Flavonoids act as negative regulators of auxin transport in vivo in Arabidopsis. Plant Physiol. 2001; 126(2): 524-535. https://doi.org/10.2307/4279917 PMID: 11402184

41. Zhou B, Wang Y, Zhan Y, Li Y, Kawabata S. Chalcone synthase family genes have redundant roles in anthocyanin biosynthesis and in response to blue/UV A light in turnip *(Brassica rapa*; Brassicaceae). AM J Bot. 2013; 100(12): 2458-2467. https://doi.org/10.3732/ajb.1300305 PMID: 24197179.

42. Han Y, Ding T, Su B, Jiang H. Genome-wide identification, characterization and expression analysis of the chalcone synthase family in maize. Int J Mol Sci. 2016; 17(2): 161. https://doi.org/10.3390/ijms17020161 PMID: 26828478.

43. Han Y, Cao Y, Jiang H, Ding T. Genome-wide dissection of the chalcone synthase gene family in *Oryza sativa*. Mol Breeding. 2017; 37(10): 119. https://doi.org/10.1007/s11032-017-0721-x.

44. Zhang Y, Hu Z, Chu G, Huang C, Tian S, Zhao Z, et al. Anthocyanin accumulation

and molecular analysis of anthocyanin biosynthesis-associated genes in eggplant (*Solanum melongena* L.). J Agr Food Chem. 2014; 62(13): 2906-2912. https://doi.org/10.1021/jf404574c PMID: 24654563.

45. Jiang M, Liu Y, Ren L, Lian H, Chen H. Molecular cloning and characterization of anthocyanin biosynthesis genes in eggplant (*Solanum melongena* L.). Acta Physiol Plant. 2016; 38(7): 163. https://doi.org/10.1007/s11738-016-2172-0

46. Wu X, Zhang A, Zhu Z, Yao J, Zha D, Li X. Effects of High ÿtemperature Stress on Active Oxygen Metabolism, Anthocyanin Content and Its Main Synthases in Eggplant Peel. Acta Ag Jiangxi. 2018; 30(06): 1-5. https://doi.org/10.19386 j.cnki.jxnyxb.2018.06.01.

Click here to access/download;Figure;Fig1.jpg

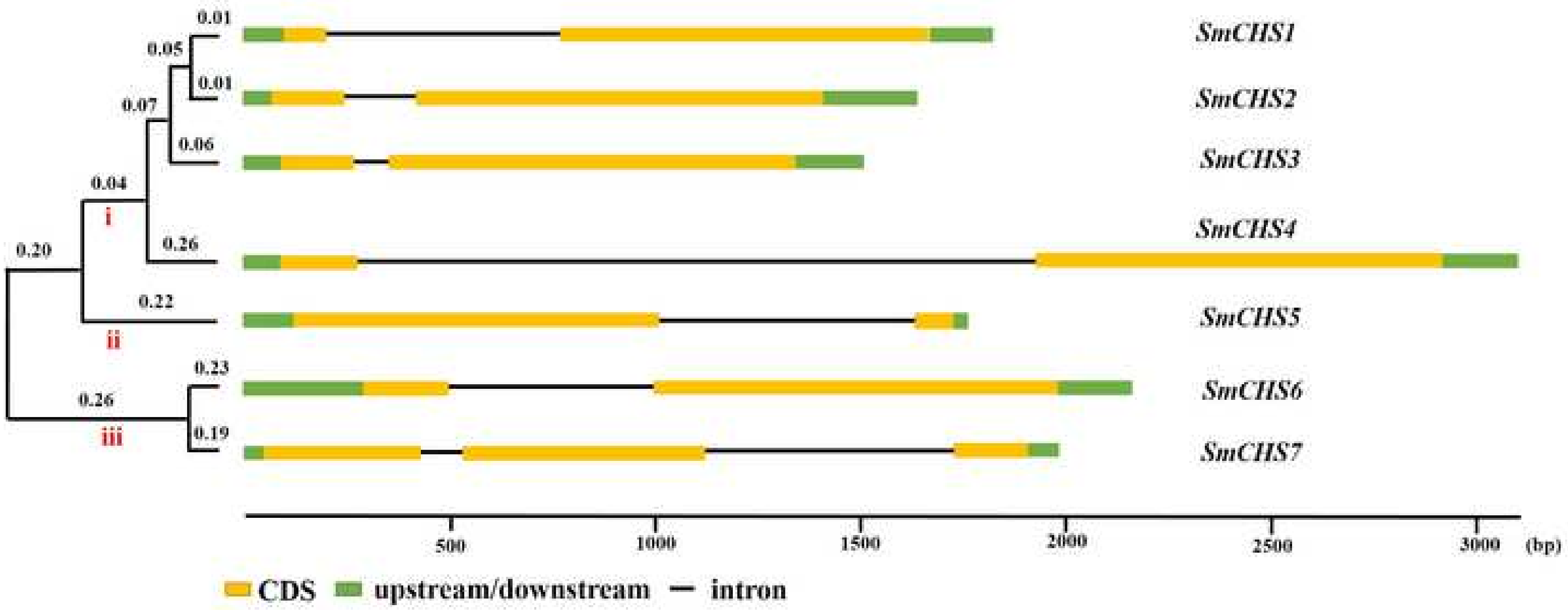

Click here to access/download;Figure;Fig2.jpg

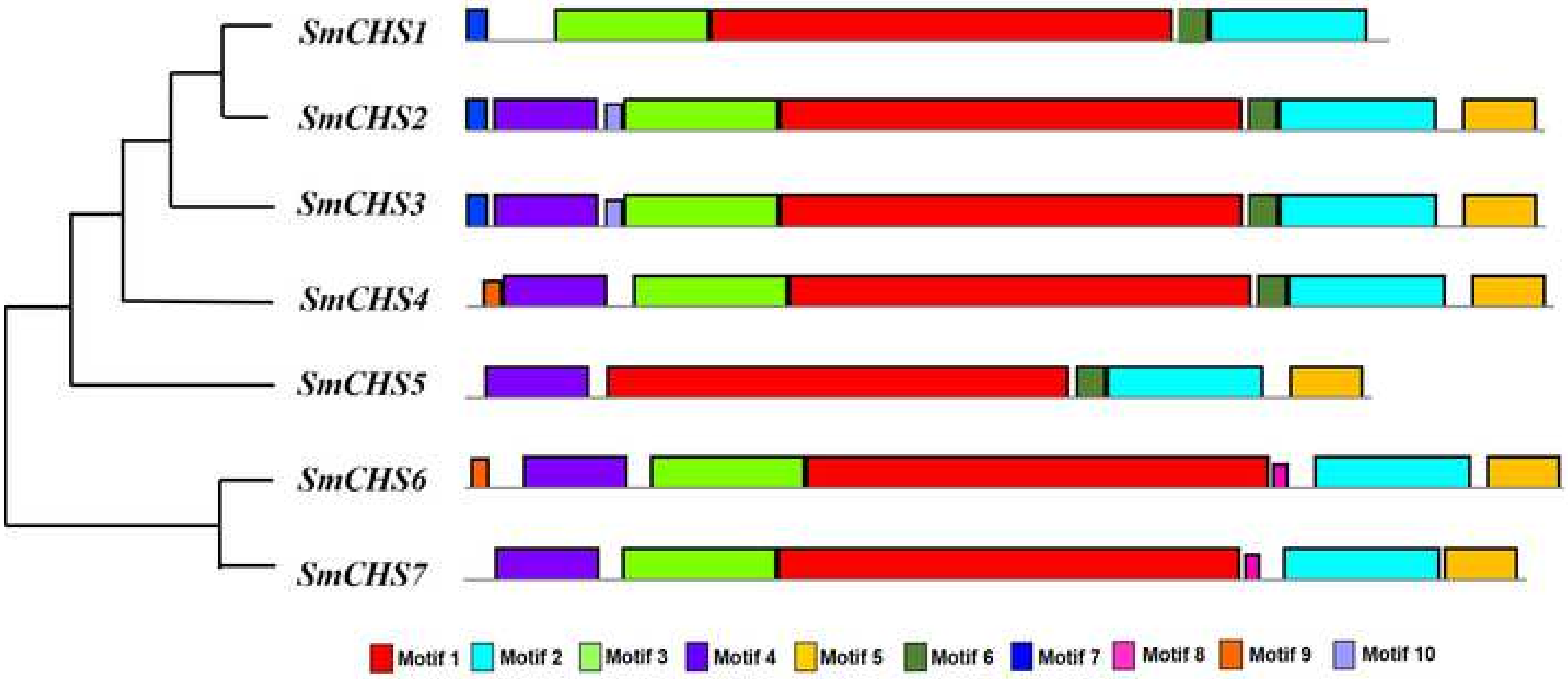

Figure

Click here to access/download;Figure;Fig5.jpg

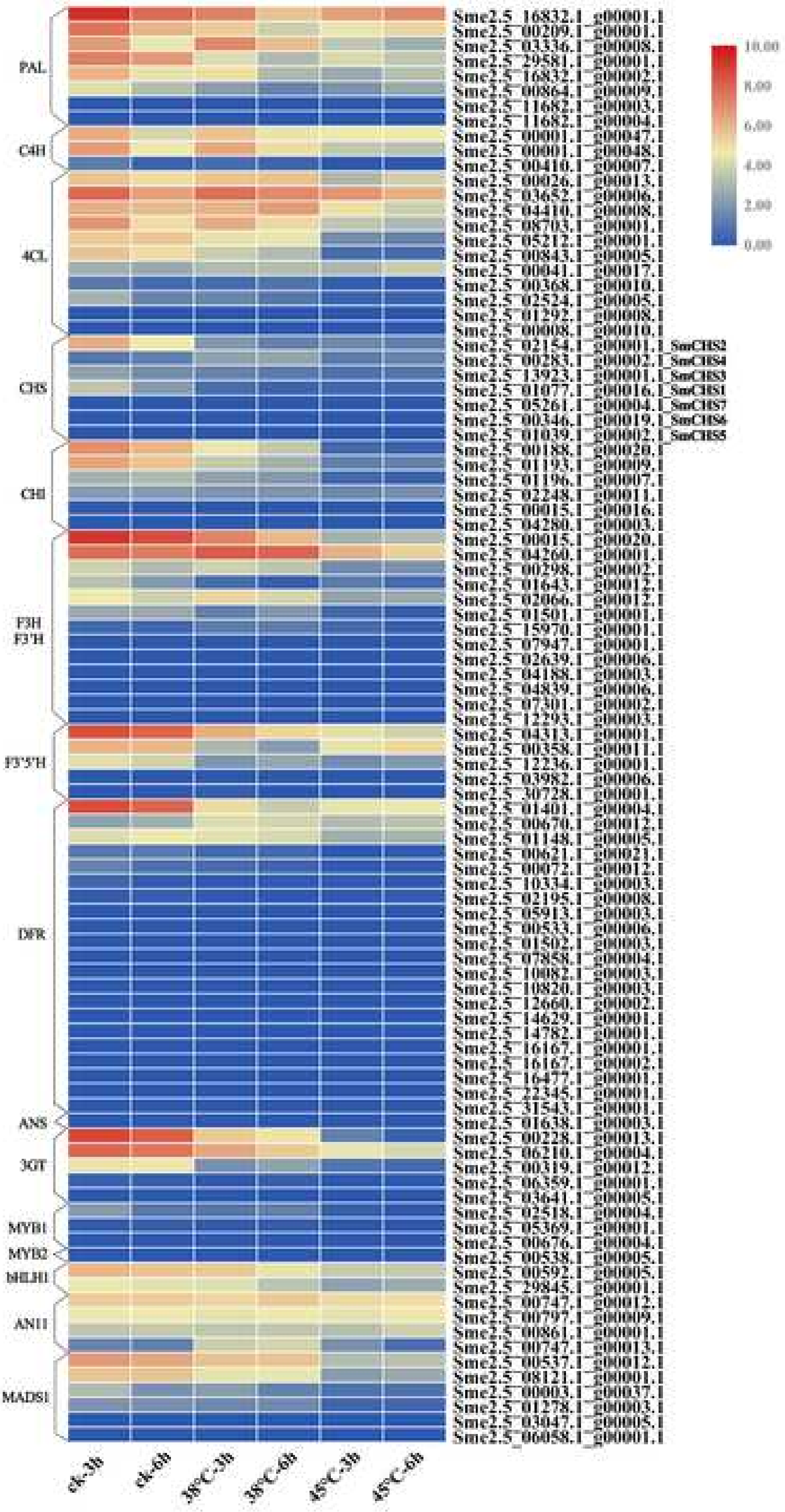

Figure

Click here to access/download;Figure;Fig3.jpg

A

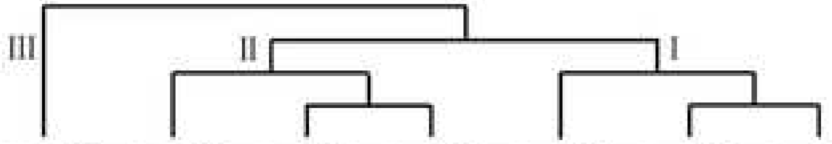

Cis-element	*SmCHS7*	*SmCHS6*	*SmCHS4*	*SmCHS5*	*SmCHS2*	*SmCHS1*	*SmCHS3*	total
ABRE	0	1	1	0	1	0	2	5
ARE	1	0	0	0	0	1	1	3
AuxRR-core	0	0	0	0	0	1	0	1
CAAT-box	12	10	17	22	14	9	16	100
CARE	0	0	0	0	1	0	0	1
CGTCA-motif	1	2	0	0	1	0	1	5
ERE	0	1	2	1	3	2	1	10
GARE-motif	1	0	0	0	0	0	0	1
G-box	0	1	0	1	0	0	1	3
GCN4-motif	0	0	0	0	0	0	1	1
GT1-motif	0	0	0	0	0	3	2	5
MBS	0	0	0	0	0	0	1	1
MRE	0	0	0	0	1	0	0	1
MYB	3	3	0	0	2	1	4	13
MYC	0	5	1	1	0	1	1	9
O2-site	0	0	0	1	1	1	0	3
STRE	2	1	1	0	0	0	0	4
TATA-box	4	12	21	21	26	16	15	115
TCA	0	2	1	1	0	0	1	5
TCT-motif	0	0	0	0	0	0	2	2
TGACG-motif	0	1	2	0	2	0	0	5
W box	0	0	1	0	2	0	1	4

B

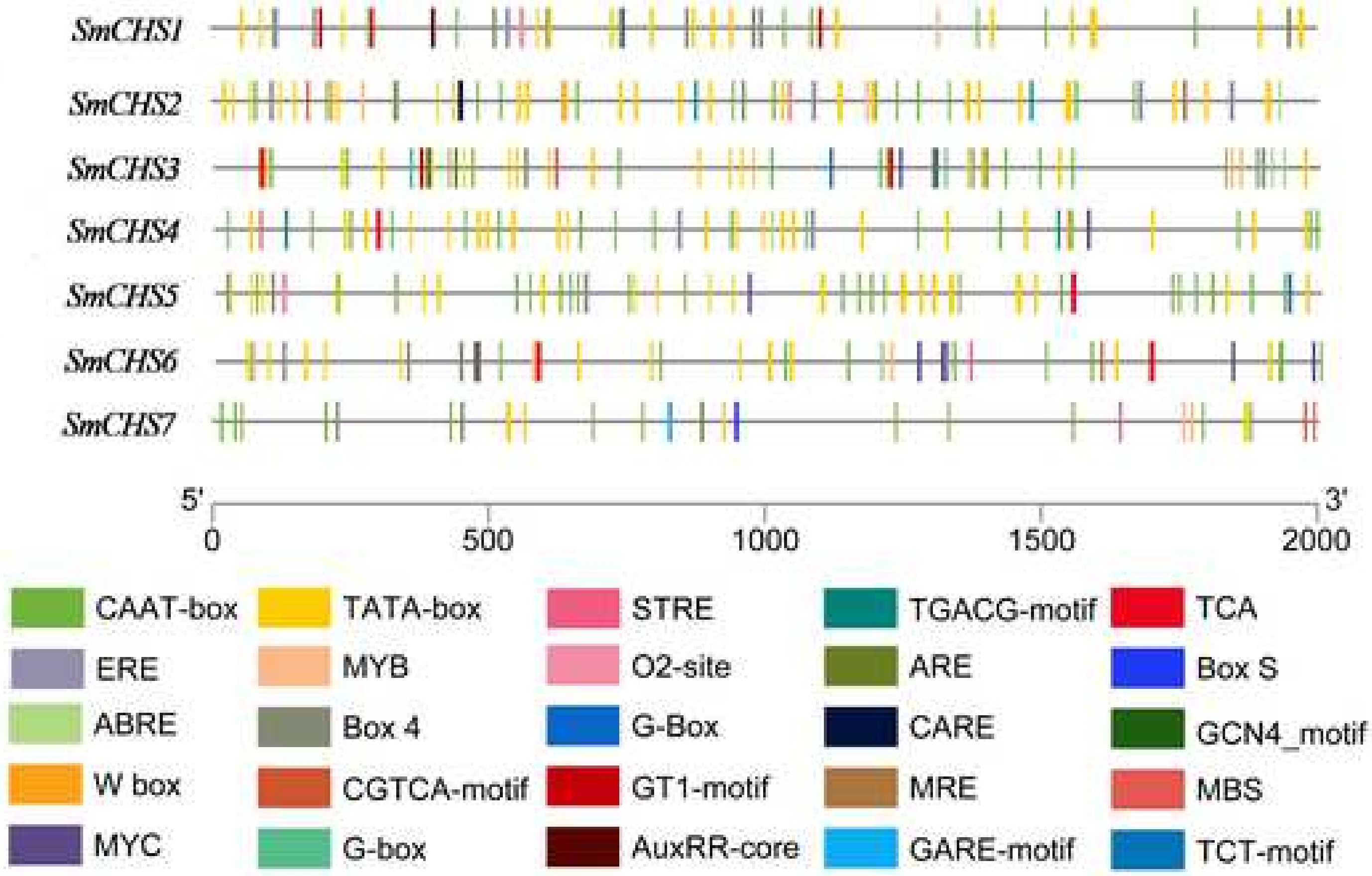

Figure

Click here to access/download;Figure;Fig4.jpg

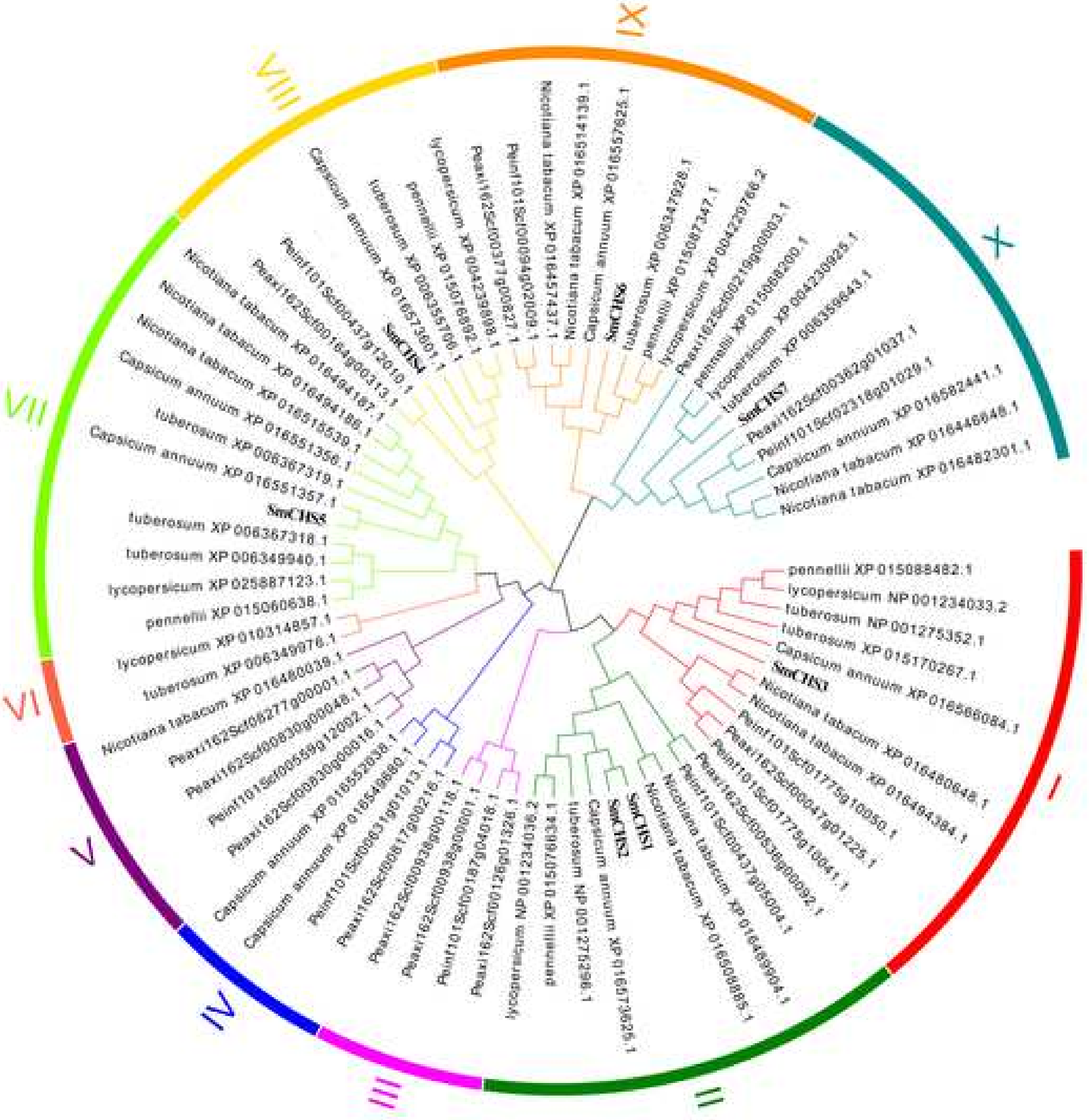

Figure

Click here to access/download;Figure;Fig6.pdf

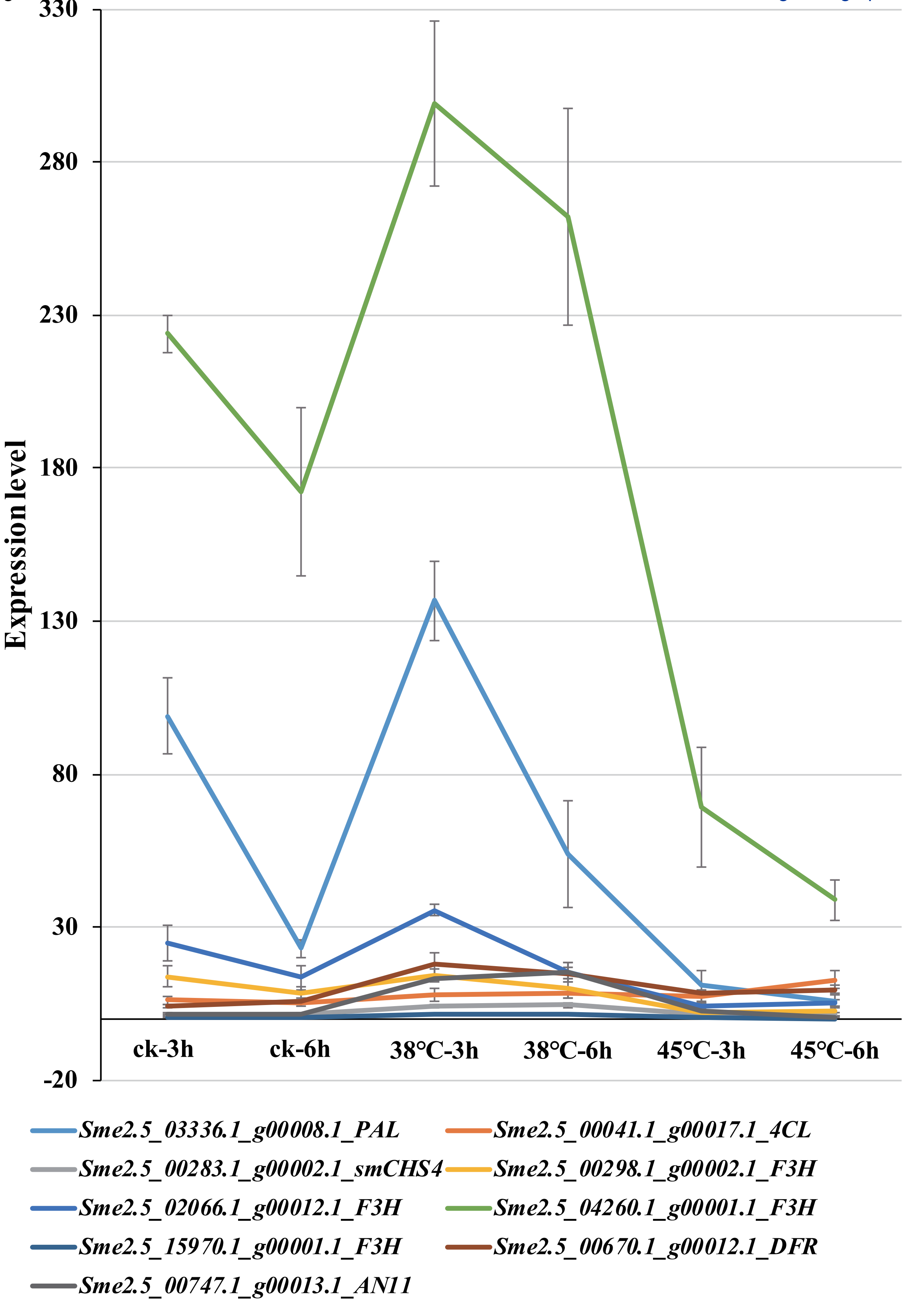

Figure

Click here to access/download;Figure;Fig7.jpg

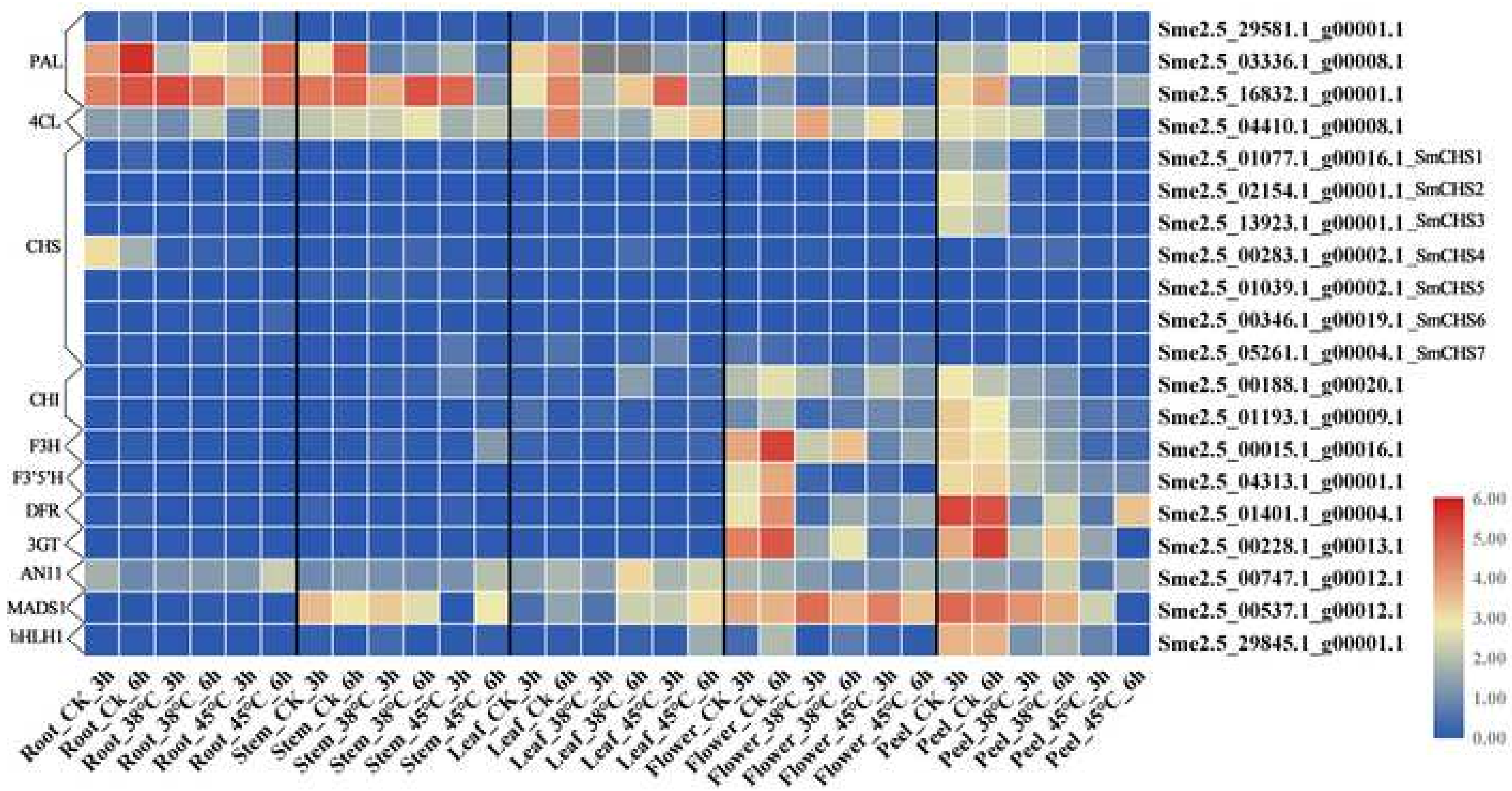

Click here to access/download
Supporting Information
S1 Fig.jpg

Click here to access/download
Supporting Information
S2 Fig.pdf

Click here to access/download
Supporting Information
S1 Table.xlsx

~~Genome-wide analysis of~~ C~~c~~halcone synthase (CHS) family members analysis from eggplant (*Solanum melongena* L.) in the flavonoid biosynthetic pathway and expression patterns in response to heat stress

Xuexia Wu¶, Shengmei Zhang¶, Xiaohui Liu, Jing Shang, Aidong Zhang, Zongwen Zhu, Dingshi Zha*

Horticultural Research Institute, Shanghai Academy of Agricultural Sciences, Shanghai Key Laboratory of Protected Horticultural Technology, Shanghai, 201403, China * Corresponding author: Dingshi Zha

E-mail: dingshizha1966@126.com

¶ These authors contributed equally to this work.

Abstract

Enzymes of the chalcone synthase (CHS) family participate in the synthesis of multiple a series of secondary metabolites in plants, fungi and bacteria. CHS showed a significant correlation with the accumulation patterns of anthocyanin. The peel color, which isprimarily determined by the content of anthocyanin, is an economically important trait for eggplants that is affected by heat stress. A total of 7 *CHS* (*SmCHS1-7*) putative genes were identified in a genome-wide analysis of eggplants (*S. melongena* L.). The *SmCHS* genes were distributed on 7 scaffolds and were classified into 3 clusters. Phylogenetic relationship analysis showed that 73 *CHS* genes from 7 Solanaceae species were classified into 10 groups. *SmCHS5*, *SmCHS6* and *SmCHS7* were continuously down-regulated under 38 ℃ and 45 ℃ treatment, while *SmCHS4* was up-regulated under 38 ℃ but showed little change at 45 ℃ in peel. Expression profiles of key anthocyanin biosynthesis gene families showed that the PAL, 4CL and AN11 genes were primarily expressed in all five tissues. The CHI, F3H, F3'5'H, DFR, 3GT and bHLH1 genes were expressed in flower and peel. Under heat stress, the expression level of 52 key genes were reduced under heat stress. In contrast, the expression patterns of eight key genes similar to *SmCHS4* were up-regulated at 38 ℃ 3h. Comparative analysis of putative CHS protein biochemical characteristics, *cis*-regulatory elements, and regulatory networks indicated that *SmCHS* gene family has a conserved gene structure and functional diversification. *SmCHS* showed two or more expression patterns and performed multiple functions to regulate anthocyanin content. Combined with analysis of regulatory networks, to the results of this study may facilitate further research to understand the regulatory mechanism governing peel color in eggplants.

Introduction

Eggplant (S. *melongena* L.) is one of the most important thermophilic vegetables produced in many tropical and temperate regions around the world. The optimum growth temperature for eggplant is between 22 and 30 °C℃. Eggplants subjected to high temperature may exhibit to stagnation of growth, abortion of flower buds, and

decrease of pollen viability rate and fruit set, and the peel's color will turn light when the temperature is over 35 °C°C. High temperature severely reduces the yield and affects the appearance quality of eggplant. However, the molecular mechanism governing high temperature stress in eggplants has not been thoroughly elucidated.

Anthocyanins are plant secondary metabolites and are among the most abundant natural pigments, that are responsible for the characteristic colors in flowers, fruits and vegetables plant tissues. The anthocyanin biosynthesis pathway has been studied in numerous plant species and most of the genes involved in this process have been identified. Moreover, anthocyanins play an important role in plant survival under stressful environmental conditions. High temperatures are known to reduce anthocyanin accumulation and have discoloration effects in many plant tissues, causing drastic effects in colored flowers [1, 2], and affecting the skin of such fruits as grape berries, apples and eggplant [3-7].

It is well known that CHS is the gatekeeper of the anthocyanin pathway [8]. Enzymes of chalcone synthase (CHS) are member of the plants-specific type III polyketide synthase (PKS) [9, 10], family and catalyze the first committed step of the branch of the phenylpropanoid pathway, which leads to the synthesis of flavonoids [11, 12]. Flavonoids are well known as a group of plant secondary metabolites that comprise several different classes of compounds, such as chalcones, flavones, flavonol isoflavones and anthocyanins. Flavonoids have a wide variety of biological functions in, for instance, flower pigmentation, protection against UV radiation, pathogen defense, auxin transport and pollen fertility [13-15]. CHS also showed a significant correlation with the accumulation patterns of anthocyanin synthesis of flavonoid compounds. during heat stress defense. –In bread wheat, heat stress responsive element has been found in the promoter of *Chs-D1* gene [16]. High-temperature stress had a large impact on the expression of *CHS7*, *CHS8* in both seeds and pods of Soybean [17]. The transcript levels of *CHS* decreased in apple peel and rose flower after heat treatment [1,

4]. In cork oak， *CHS* gene expression exhibited an increase under 45 ℃, but showed a decreased expression at 55 ℃[18]. The emergence of *CHSV* and *CHSVII* is important for the development of fungal heat stress tolerance and pathogenicity in pathogenic fungi. [19]. In addition, *CHS* (Sme2.5_00283.1_g00002.1) was up-regulated, and the other two *CHS* gene members were down-regulated under heat stress in peel of eggplant [7].

~~. After heat treatment, the transcript levels of *CHS* decreased in the rose flower and in the eggplant [1, 7].~~

The product of the CHS reaction is a pivotal precursor for a large array of secondary metabolites derived from malonyl-CoA and p-coumaroyl-CoA. CHS exists as homodimeric iterative PKS (monomer size of 42––45 kDa) with two independent active sites that catalyze a series of decarboxylation, condensation, and cyclization reactions [10, 20]. Member of the CHS superfamily share high similarity in their amino acid sequence, which contains the structurally conserved catalytic center consisting of four residues, Cys-His-Asn-Phe, and most of the genes contain two exons and one intron [21]. However, the *CHS* gene family has not been characterized in eggplants to date.

~~Anthocyanins are plant secondary metabolites and are among the most abundant natural pigments, that are responsible for the characteristic colors in flowers, fruits and vegetables plant tissues. The anthocyanin biosynthesis pathway has been studied in numerous plant species and most of the genes involved in this process have been identified. The enzymes evolved in anthocyanin biosynthesis are as follows: phenylalanine ammonia lyase (PAL), cinnamate 4-hydroxylase (C4H), 4-coumarateCoA ligase (4CL), chalcone synthase (CHS), chalcone isomerase (CHI), flavanone 3-hydroxylase (F3H), flavonoid 3′-hydroxylase (F3′H), flavonoid 3′5′-hydroxylase (F3′5′H), dihydroflavonol4-reductase (DFR), anthocyanidin synthase (ANS), and anthocyanidin 3-O-glucosyltransferase (3GT). Most transcription factors, such as myeloblastosis (MYB) and basic helix-loop-helix (bHLH), are positive regulators of anthocyanin biosynthesis in vegetative tissues. The production of chalcone requires the condensation of one molecule of p-coumaroyl-CoA and three malonyl-CoA molecules which is catalyzed by CHS. Taken together, these findings~~

~~indicate that CHS is the gatekeeper of the anthocyanin pathway.~~

~~Anthocyanins play an important role in plant survival under stressful environmental conditions. High temperatures are known to reduce anthocyanin accumulation and have discoloration effects in many plant tissues, causing drastic effects in colored flowers, and affecting the skin of such fruits as grape berries, apples and eggplant.~~

~~Eggplant (S. *melongena* L.) is one of the most important thermophilic vegetables produced in many tropical and temperate regions around the world. The optimum growth temperature for eggplant is between 22 and 30°C. Eggplants subjected to high temperature may exhibit to stagnation of growth, abortion of flower buds, and decrease of pollen viability rate and fruit set, and the peel's color will turn light when the temperature is over 35°C. High temperature severely reduces the yield and affects the appearance quality of eggplant. However, the molecular mechanism governing high temperature stress in eggplants has not been thoroughly elucidated.~~

In the current study， all *SmCHS* family members were identified in eggplant. A comprehensive analysis of members was performed, including gene structures, the biochemical characteristics of putative CHS protein, promoter *cis*-elements, phylogenetic relationships among members in other relative species, and their expression profiles in various organs/tissues under high temperature stress. The findings of the present study may facilitate functional studies on eggplant *SmCHS* family genes.

Materials and methods

Plant materials and RNA extraction

The eggplant cultivar 'Tewangda' is a cold-tolerant cultivar with blackish purple skin. This cultivar grows vigorously and has good fruit setting. The fruit shape has a 27.6 ~~-~~cm fruit length, a 5.4 ~~-~~cm transverse diameter and a 209 ~~-~~g single fruit weight on average. The 'Tewangda' fruit has good commercial properties and good transportation resistance. 'Tewangda' fruits were grown at the same growth stage and were randomly selected. These plants were grown 144 days after sowing, and then placed inside

incubators set at 27 ℃ (CK), 38 ℃ or 45 ℃ for 3 or 6 h (three plants per treatment). For each treatment, the tissue samples of root, stem, leaf, flower and peel were obtained and immediately frozen in liquid nitrogen and stored at -80 °C for RNA extraction and other analyses. All plant materials examined in this study were obtained from Shanghai Academy of Agricultural Sciences. Total RNA was extracted from each tissue sample using the mirVana miRNA Isolation Kit (Ambion) following the manufacturer's protocol. The extracted total RNA was stored at -80 °C. RNA integrity was evaluated using the Agilent 2100 Bioanalyzer (Agilent Technologies, Santa Clara, CA, USA).

Identification of the CHS family members in the eggplant genome

The whole protein sequence of *Solanum melongena* L. (eggplant) were obtained from the Eggplant Genome DataBase (http://eggplant.kazusa.or.jp) [22], and those of *Solanum tuberosum* L. (potato, http://solanaceae.plantbiology.msu.edu/pgsc_download.shtml) [23], *Solanum lycopersicum* (tomato, https://solgenomics.net/organism/Solanum_lycopersicum/genome) [24], *Solanum penellii* (wild tomato, https://www.plabipd.de/project_spenn/start.ep) [25], *Capsicum annuum* L. (pepper, http://peppergenome.snu.ac.kr) [26], *Petunia axillaris* (https://solgenomics.net/organism/Petunia_axillaris/genome) [27], *Petunia inflate* (https://solgenomics.net/organism/Petunia_inflata/genome) [27], and *Nicotiana tabacum* (common tobacco, https://www.ncbi.nlm.nih.gov/nuccore/AYMY00000000) [28]. The profiles of CHS (PF00195 and PF02797) wwere downloaded from the Pfam protein family database (http://pfam.xfam.org/), and these profile sequences were used as queries to perform BLASTP searches against the protein sequence data of all the species mentioned above with a maximum E-value of 1×10^{-3}, respectively [29]. To further verify the exact copy number of CHS and remove redundant sequences, the Pfam database and Genome websites were also searched using "chalcone synthase" as keywords. All CHS sequences were submitted to EXPASy

(https://web.expasy.org/protparam/) to calculate the number of amino acids, molecular weights and theoretical isoelectric points (pI).

Structural characterization

The locations and intron numbers of CHS were acquired through the genome website. All of the acquired protein sequences were first aligned by ClustalX software with the default parameters [30]. An unrooted maximum-likelihood phylogenetic tree was constructed using MEGA6 software with a bootstrap test of 1000 times [31]. The MEME program (Version 5.0.5, http://meme-suite.org/tools/meme) was used to identify the conserved motif of the CHS sequences with the following parameters: any number of repetitions, maximum of 10 misfits and optimum motif width of 6-200 amino acid residues. The WoLF PSORT program was used to predict the subcellular localization information of CHS proteins (https://www.genscript.com/wolf-psort.html) [32].

Analysis of *cis*–acting elements in SmCHS

The upstream sequences (2 kb) of the *SmCHS* coding sequences in eggplant were retrieved from the genome sequence and then submitted to PlantCARE (http://bioinformatics.psb.ugent.be/webtools/plantcare/html/) to identify regulatory elements [33].

Phylogenetic analysis of CHS genes

The full-length protein sequences of all eight species in Solanaceae were used for phylogenetic analysis. All of the protein sequences were first aligned by ClustalX software with the default parameters [30]. The phylogenetic tree was generated with MEGA6 software with a bootstrap test of 1000 times. The final tree was viewed and modified in Evolview software [34]. The CHS genes were classified into different groups according to the topology of the phylogenetic tree.

Expression analysis of antyocyanin biosynthetic genes and ~~C~~construction of the mRNA regulatory network

The RNA-seq results were obtained by our lab [35]. Gene expression level was estimated from mean FPKM (fragments per kilobase of exon model per million reads mapped) values for each treatment, and showed the expression patterns in heatmap. Significant differentially expressed genes (fold change ≥ 2 and *p*-value ≤ 0.05) were used to calculate the Pearson correlation coefficient between *CHS* genes and other genes. The TBtools program was used to elucidate the Gene Ontology (GO) functional classification for the mRNAs with correlation coefficients greater than 0.9 [36]. The top 5 regulatory mRNAs annotated by GO enrichment for the genes associated with anthocyanin biosynthesis were collected to construct the regulatory network. The network was visualized using Cytoscape [37].

qRT-PCR analysis

Expression level of anthocyanin biosynthesis genes, including phenylalanine ammonia lyase (PAL), cinnamate 4-hydroxylase (C4H), 4-coumarateCoA ligase (4CL), CHS, chalcone isomerase (CHI), flavanone 3-hydroxylase (F3H), flavonoid 3′-hydroxylase (F3′H), flavonoid 3′5′-hydroxylase (F3′5′H), dihydroflavonol4-reductase (DFR), anthocyanidin synthase (ANS), anthocyanidin 3-O-glucosyltransferase (3GT), Anthocyanins11 (AN11), and most transcription factors, such as myeloblastosis (MYB), basic helix-loop-helix (bHLH) and metadata authority description schema(MADS1), were analyzed. First-strand cDNA was synthesized from 1 μg from 5 tissues (root, stem, leaf, flower and peel) using a Prime Script RT Reagent Kit (Takara, Dalian, China). The qRT-PCR reactions were performed in 96-well plates using the ABI 7500 fast Real-Time PCR system (Applied Biosystems, USA) with the QuantiFast SYBR Green PCR Kit (Qiagen, Duesseldorf, Germany). The qRT-PCR parameters were as follows: 95 ℃ for 5 min, then 45 cycles of 95 ℃ for 10 s, 60 ℃ for 10 s, and 72 ℃ for 10 s. The relative mRNA expression levels were calculated using the $2^{-\Delta\Delta CT}$ method [38]. PGK(JX154676) was used as an internal control to normalize the data. For each sample, three biological

repeats were performed, the relative expression levels were calculated using the standard curve and normalized by the control's expression, the results were display by heatmap. The primer sequences are listed in Additional file 3 Table S1S3.

Results

Identification of *CHS* genes and sequence analysis in Solanaceae species

A total of 7 *CHS* (*SmCHS1-7*) genes in eggplant were identified after being verified by protein sequence analysis and BlAST search using the eggplant genome annotation database (Additional file 1 Table S1a). The length of SmCHS protein ranged from 327 to 396 amino acids (Table 1, Table S2). The PKS type III active sites of the enzymes and Phe215 connected with CoA binding are conserved among all SmCHS (S1 Fig). The molecular weights of SmCHS were between 35.2 kDa and 43.7 kDa. The theoretical pI value of SmCHS ranged from 5.59 to 7.04. In addition, 66 *CHS* genes were characterized from 7 other Solanaceae species. The subfamily numbers of *CHS* genes ranged from 6 (*Solanum penellii*) to 13 (*Petunia axillaris*) (Table 1, Additional file 1 Table S1b-h). The molecular weights of CHS for the other 7 Solanaceae species ranged from 17.3 to 47.4, the length for the other 7 Solanaceae speciesof proteins ranged from 156 to 431 amino acids, and the theoretical pI value ranged from 5.1 to 8.47 (Additional file 2 Table S1a-g). The average number of amino acids, molecular weight and theoretical pI were was calculated and then employed as a data set for each species. The correlation coefficients among the above data were all greater than 0.99. This finding suggests that *CHS* genes are conserved in Solanaceae species.

Table 1. Features of *SmCHS* genes identified in eggplant.

Gene Name	Gene ID	Number of amino acids
SmCHS1	Sme2.5_01077.1_g00016.1	333
SmCHS2	Sme2.5_02154.1_g00001.1	389
SmCHS3	Sme2.5_13923.1_g00001.1	389
SmCHS4	Sme2.5_00283.1_g00002.1	392

SmCHS5	Sme2.5_01039.1_g00002.1	327
SmCHS6	Sme2.5_00346.1_g00019.1	396
SmCHS7	Sme2.5_05261.1_g00004.1	383

Structure and conserved motif analysis of *SmCHS*

The 7 *SmCHS* genes were distributed on 7 scaffolds. To better understand the evolution of *SmCHS* genes, an unrooted maximum-likelihood tree was constructed based on the 7 SmCHS protein sequences, and the *SmCHS* were classified into 3 clusters (i, ii and iii) (Fig 1). Among the *SmCHS* genes, only one *SmCHS7* had three exons, and the others had two exons (Fig 1) based on information available from the genome annotation. These results suggest the potential diversity of the biological functions of the *SmCHS* genes in eggplants.

Fig 1. Phylogenetic relationship and gene structure analysis of *SmCHS* genes.

The phylogenetic tree was on the left of the figure and showed that the *SmCHS* genes were classified into three clusters (i, ii and iii). The exon/intron organization of *SmCHS* is shown on the right of the figure. For *SmCHS* gene organization, yellow boxes represent exons, black lines represent introns, and green boxes indicate upstream/downstream regions. The lengths of the exons and introns are drawn to scale.

To understand the functional diversification of *SmCHS*, the conserved motifs of these 7 protein sequences were identified by the MEME program, and 10 conserved motifs were detected in eggplant (Fig 2, Table 2). The Chal_sti_synt_C domain and Chal_sti_synt_N domain were included in motifs 1 and motifs 2, respectively. For all 7 eggplant SmCHS proteins, Motif 1 and ~~M~~motif 2 exist in all of them, m~~M~~otif 3 is only absent in *SmCHS5*, and m~~M~~otif 4 and m~~M~~otif 5 are only absent in *SmCHS1*. The N-terminal domain (PF00195) of the CHS protein contained m~~M~~otif 1 and the combination of m~~M~~otifs 3, 4, 6, 7 and 9. The C-terminal domain (PF02797) of the CHS protein contained m~~M~~otif 2 and the combination of m~~M~~otifs 5, 8 and 10. Therefore, the

motif configuration of the SmCHS reflects the conservation and diversity of the CHS family. To further investigate the subcellular localization information of SmCHS proteins, the WoLF PSORT program was used to predict the localization of SmCHS protein [31]. SmCHS7 was predicted to localize in the nucleus, and SmCHS4 and SmCHS6 were predicted to localize in the chloroplast. The others SmCHS proteins were predicted to localize in the cytoplasmic. The different compositions of the domains and subcellular localization may indicate functional diversity.

Table 2 List of the putative motifs of CHS proteins

Motif	Width	Best possible match
Motif 1	167	IKEWGQPKSKITHLVFCTTSGVDMPGADYQLTKLLGLRPSVKRFMMYQQGCFAGGTVLRLAKDLAENNKGARVLVVCSEITAVGFRGPSETHPDSLVGQA
Motif 2	57	DWNSJFWIAHPGGPAILDQVELKLGLKPEKLRATRQVLSDYGNMSSACVLFILDEMR
Motif 3	56	RLCDKSMIKKRYMHLTEEILKENPNLCEYMAPSLDARQDIVVVEVPKLGKEAAQKA
Motif 4	38	QRAEGPATILAIGTATPSNCVDQSTYPDYYFRITNSEH
Motif 5	27	TTGEGLDWGVLLGFGPGLTIETIVLHS
Motif 6	11	LIEAFEPLGIS
Motif 7	8	MVTVEEVR
Motif 8	6	FCEKLI
Motif 9	7	QNIGKVN
Motif 10	7	ELKEKFK

Fig 2. Motifs conserved across all CHS proteins in eggplant. Ten conserved motifs are indicated in differently colored boxes.

Stress-related *cis*-elements in *SmCHS* promoters

To further study the potential regulatory mechanisms of *SmCHS* during abiotic stress responses, the 2-kb upstream sequences from the translation start sites of *SmCHS* were used to identify the *cis*-elements (Fig 3B). The results showed that all *SmCHS* had common upstream promoter elements, including TATA-box and CAAT-box, which occurred more than 100 times; therefore, these sequences were presumed to be the

promoter sequences (Fig 3A). The elicitor response (ERE) and myeloblastosis (MYB) occurred more than 10 times in the *SmCHS* upstream sequences. Research has shown that an increase in CHS activity causes a high accumulation of flavonoids that inhibits polar auxin transport [8, 39, 40]. Two *cis*-acting elements (ABRE, involved in abscisic acid responsiveness; AuxRR, involved in auxin responsiveness) were found in the upstream regions. MYB and myelocytomatosis (MYC) binding sites have also been identified, which may greatly influence plant stress tolerance. Cluster analysis of *cis*-element number showed that 7 *SmCHS* genes were divided into 3 groups (Ⅰ, Ⅱ, Ⅲ), and *SmCHS1*, *SmCHS2* and *SmCHS3* had similar regulatory pattern (Fig 3A). Five *cis*-elements (CARE, GCN4-motif, GT1-motif, MRE and TCT-motif) exist only in group Ⅰ, GARE-motif only exist in group Ⅲ. STRE exist in group Ⅱ and Ⅲ. These results showed that *SmCHS* is activated by a wide range of environmental and developmental stimuli, and there are many complex means of regulating *SmCHS* activity in eggplants.

Fig 3. *Cis*-elements in *CHS* family gene promoters. (A) Frequency of cis-element occurrence in upstream sequences. (B) Predicted *cis*-elements in *CHS* gene promoters. The scale bar indicates the length of promoters.

Phylogenetic analysis of *CHS* genes in Solanaceae

To analyze the evolutionary relationships of *CHS* genes in Solanaceae, an unrooted phylogenetic tree was constructed using full-length amino acid sequences. All 73 *CHS* genes were classified into 10 groups (Fig 4, Table 3), and the number of CHS gene groups ranged from two to eleven. The 7 SmCHS ~~genes~~ were categorized into 6 groups (groups Ⅰ, Ⅱ, Ⅶ, Ⅷ, Ⅸ and Ⅹ), and group Ⅱ contained *SmCHS1* and *SmCHS2*. Groups Ⅰ, Ⅱ, Ⅸ and Ⅹ exist in all eight species, and groups Ⅲ, Ⅳ and Ⅴ were absent in *Solanum melongena* L., *Solanum penellii*, *Solanum lycopersicum* and *Solanum tuberosum* L.. The group Ⅵ is absent in *Capsicum annuum* L., *Nicotiana tabacum*, *Petunia inflate* and *Petunia axillaries* (Table 3). The Ⅷ, Ⅸ and Ⅹ groups are distinguished from other

Formatted: Font: Not Italic

groups mainly depends on the position 1-164 amino acids, Groups Ⅰ, Ⅱ and Ⅲ are relatively conservative at the position 260-360 amino acids, in which the other groups are very diverse(S1 Fig). These results suggested that the CHS ~~genes~~ were conserved, but small variations existed among the eight species in Solanaceae and showed that SmCHS1, SmCHS2 and SmCHS3 were more conserved than *SmCHS4* according to the phylogenetic tree.

Table 3. Distribution of *CHS* genes in the phylogenetic tree.

Plant Pecies	Number	Phylogenetic Group									
		Ⅰ	Ⅱ	Ⅲ	Ⅳ	Ⅴ	Ⅵ	Ⅶ	Ⅷ	Ⅸ	Ⅹ
Solanum melongena L.	7	1	2	0	0	0	0	1	1	1	1
Solanum penellii	6	1	1	0	0	0	0	1	1	1	1
Solanum lycopersicum	7	1	1	0	0	0	1	1	1	1	1
Solanum tuberosum L.	10	2	1	0	0	0	1	3	1	1	1
Capsicum annuum L.	9	1	1	0	2	0	0	2	1	1	1
Nicotiana tabacum	12	2	2	0	0	1	0	3	0	2	2
Petunia inflate	9	2	1	1	1	1	0	0	1	1	1
Petunia axillaris	13	1	1	3	1	3	0	0	1	1	2

Fig 4. Phylogenetic tree of *CHS* genes in Solanaceae species. The color region is associated with 10 groups of proteins (Group Ⅰ to Ⅹ).

Expression profile of key anthocyanin biosynthesis genes in eggplants under heat stress

Using the RNA-seq data, a heatmap of 96 key anthocyanin biosynthesis genes (PAL, C4H, 4CL, CHS, CHI, F3H/F3'H, F3'5'H, DFR, ANS, 3GT, MYB1, MYB2, bHLH1, AN11, MADS1) was established under heat stress (Fig 5). The expression of anthocyanidin synthase (ANS) and MYB2 was not identified during this sampling period. For seven *SmCHS* genes, three (*SmCHS5*, *SmCHS6*, and *SmCHS7*) were not identified, and the other four *SmCHS* genes were divided into two groups according to their expression patterns. Three of those four *SmCHS* genes (~~*SmCHS5*~~*SmCHS1*, ~~*SmCHS6*~~*SmCHS2*, and ~~*SmCHS7*~~*SmCHS3*) were continuously down-regulated under

Formatted: Font: Not Italic

Formatted: Font: Not Italic

Formatted: Font: Not Italic

Formatted: Font: Not Italic

38 ℃ and 45 ℃ treatment compared with the CK. However, *SmCHS4* was up-regulated under 38 ℃, but showed little change at 45 ℃ in peel. These phenomena have also been observed in some other key gene families associated with anthocyanin biosynthesis. According to the RNA-seq results of 96 anthocyanin biosynthesis key genes in eggplant peel, *SmCHS4* showed the highest expression level at the 38 ℃-3h along with eight other genes (*Sme2.5_03336.1_g00008.1_PAL*, *Sme2.5_00041.1_g00017.1_4CL*, *Sme2.5_00283.1_g00002.1_smCHS4*, *Sme2.5_00298.1_g00002.1_F3H*, *Sme2.5_02066.1_g00012.1_F3H*, *Sme2.5_04260.1_g00001.1_F3H*, *Sme2.5_15970.1_g00001.1_F3H*, *Sme2.5_00670.1_g00012.1_DFR*, *Sme2.5_00747.1_g00013.1_AN11*) (Fig 6~~7~~). In particular, *Sme2.5_03336.1_g00008.1_PAL* expression level under 38 ℃ doubled but was down-regulated at 45 ℃ compared with CK; *Sme2.5_00670.1_g00012.1_DFR*, *Sme2.5_00747.1_g00013.1_AN11* expression level increased 3-4 -fold and 7-10 -fold under 38 ℃, respectively.

Fig 5. ~~Expression profile~~Heatmap of 96 key anthocyanin biosynthesis genes expression level in eggplants peel under heat stress. The color box from blue to red indicate an increased expression level.

Fig ~~7~~6. Expression profiles of *SmCHS4* and ~~Key~~eight anthocyanin biosynthesis genes ~~expression profiles~~ in response to heat stress. These genes have the highest expression level at 38 ℃-3h in eggplant peel. ~~were observed at the highest expression level at 38 ℃ 3h.~~ The error bars represent the standard error of the means of three biological replicates.

mRNA regulatory network associated with anthocyanin biosynthesis in eggplant

A total of 4928 mRNA correlation coefficients were more than 0.9, and all of these mRNAs were functionally categorized in the GO database. The top 20 GO enrichment

results of biological processes are shown in Table 4. The function was involved in the regulation of biological processes (GO:0050789), regulation of cellular metabolic processes (GO:0031323) and regulation of gene expression (GO:0010468) were collected and filtered to construct a regulatory network. In totally, 67 anthocyanin biosynthesis key genes and 146 regulatory mRNAs were included in this regulatory network (~~S1~~ S2 Fig). These GO enrichment results suggest that the anthocyanin biosynthesis pathway may be regulated by a wide range of environmental and developmental stimuli.

Table 4. Top 20 GO enrichment results of biological processes.

GO term	GO ID	P value
cellular biosynthetic process	GO:0044249	0
cellular nitrogen compound biosynthetic process	GO:0044271	0
cellular response to chemical stimulus	GO:0070887	0
cellular response to stress	GO:0033554	0
regulation of biological process	GO:0050789	0
regulation of cellular macromolecule biosynthetic process	GO:2000112	0
developmental process	GO:0032502	0
regulation of RNA biosynthetic process	GO:2001141	0
regulation of cellular metabolic process	GO:0031323	0
cellular component organization	GO:0016043	0
response to organic substance	GO:0010033	1.11E-16
protein metabolic process	GO:0019538	1.11E-16
regulation of nitrogen compound metabolic process	GO:0051171	1.11E-16
regulation of gene expression	GO:0010468	1.11E-16
response to stimulus	GO:0050896	1.11E-16
regulation of nucleobase-containing compound metabolic process	GO:0019219	1.11E-16
response to chemical	GO:0042221	1.11E-16
cell communication	GO:0007154	1.11E-16
response to stress	GO:0006950	1.11E-16
oxoacid metabolic process	GO:0043436	2.22E-16

Expression pattern of anthocyanin biosynthesis key genes in different tissues under heat stress

Using the qRT-PCR data, a heatmap of 20 key anthocyanin biosynthesis genes was

established in different tissues under heat stress (Fig 76). The qRT-PCR results showed a high consistency with the RNA-seq data, which suggested that the RNA-seq data were credible. Most of the CHS genes were expressed in peel and were expressed at low levels in other tissues. The PAL, 4CL and AN11 genes were mainly expressed in all five tissues. The CHI, F3H, F3’5’H, DFR, 3GT and bHLH1 genes were expressed in flower and peel. MADS1 was expressed in stems, leaves, flowers and peels. Under heat stress， cluster i (cluster show in Fig 2) was continuously downregulation , cluster ii was up-regulated 4 times under 38 ℃ compared with CK in peel, and cluster iii was not detected in most eggplant tissues.

Fig 67. Expression profiles of 20 key anthocyanin biosynthesis genes in different tissues.

Discussion

It is well-known that the *CHS* gene family plays a significant role in the growth and development of plants. In many species, multigene families of *CHS* have been identified. For example, six *CHS* genes have been described in turnip [41]. In maize, 14 complete *CHS* genes have been identified [42]. A total of 27 *CHS* genes were found in rice [43]. These studies showed that *CHS* members were divided into two or more subclasses according to phylogenetic analysis. Generally, genes grouped into the same subclasses shared similar evolutionary features, and obtained the same expression pattern. In our study, the identified sequences showed a high level of coding sequence similarity (above 90%). The *SmCHS* were classified into three clusters based on the results of the maximum-likelihood tree. Under heat stress， cluster i was continuously downregulation , cluster ii was upregulated 4 times under 38℃ compared with CK in peel, and cluster iii was not detected in most eggplant tissues. At 35 ℃, previous studies showed that *SmCHS1* and *SmCHS3* (Sme2.5_01077.1_g00016.1, Sme2.5_13923.1_g00001.1) were down-regulated in peels of eggplant [7](Lv et al. 2019), which is in keeping with our results, other two clusters *CHS* genes show different

Formatted: Font: Italic

expression patterns. These results suggest the functional diversification of *SmCHS*.

Flavonoids have numerous functions and contribute to pigments, signaling molecules, and protectants against biotic and abiotic stresses. The flavonoid biosynthetic pathway is one of the most intensively investigated pathways for applied biological and genetic processes, as well as for understanding gene regulation, characterizing transposable elements and producing of agronomically stress-tolerant plants and natural dietary antioxidants. biosynthesis of anthocyanins responds to environmental stressors, such as light, nutrient depletion, and temperature change. The peel color determined by the content of anthocyanin is ~~an~~a majority economically important trait for eggplant, and this color is modulated by the genes in the flavonoid biosynthesis pathway. Compared with other tissues, *SmMYB1* and all anthocyanin biosynthetic key genes (*SmCHS*, *SmCHI*, *SmF3H*, *SmDFR*) except *SmPAL* were dramatically up-regulated in the fruit skin of the purple cultivar [44]. The full length cDNA of *SmCHS*, *SmCHI*, *SmF3'5'H*, and *SmDFR* were isolated from eggplants by Jiang. These genes have the highest expression levels in peels except for *SmF3H*, which was detected in stems [45]. The expression profiles of these key gene families under heat stress were investigated in our study. ~~The PAL, 4CL and AN11 genes were mainly expressed in all five tissues. The CHI, F3H, F3' '5' H, DFR, 3GT and bHLH1 genes were expressed in the flower and peel~~These anthocyanin biosynthesis key genes (PAL, 4CL, AN11, CHI, F3H, F3'5'H, DFR, 3GT and bHLH1) show tissue specific expression, suggesting that these genes respond at the late stage of the anthocyanin pathway and directly regulate the color of fruit skin and flower.

Heat stress reduced the anthocyanin content and the enzyme activities of CHS, DFR, ANS and 3GT/UFGT in eggplant peel and strengthened the activity of PAL [35, 46]. When the temperature exceeds 35 ℃, the eggplant will be dehydrated and shrink, and the peel's color will lighten. CHS is a key enzyme of the flavonoid biosynthesis pathway. Most of the genes associated with flavonoid biosynthesis were down-regulated under heat stress. In this study, the genes of the flavonoid biosynthesis pathway showed tissue-

specificity, and genes expressed in different phases and tended to change over time (Fig 7~~6~~). ~~According to the RNA-seq results of 96 anthocyanin biosynthesis key genes in eggplant peel, *SmCHS4* showed the highest expression level at the 38℃-3h along with eight other genes (*Sme2.5_03336.1_g00008.1_PAL*, *Sme2.5_00041.1_g00017.1_4CL*, *Sme2.5_00283.1_g00002.1_smCHS4*, *Sme2.5_00298.1_g00002.1_F3H*, *Sme2.5_02066.1_g00012.1_F3H*, *Sme2.5_04260.1_g00001.1_F3H*, *Sme2.5_15970.1_g00001.1_F3H*, *Sme2.5_00670.1_g00012.1_DFR*, *Sme2.5_00747.1_g00013.1_AN11*) (Fig 7). In particular, *Sme2.5_03336.1_g00008.1_PAL* expression level under 38℃ doubled but was downregulated at 45℃ compared with CK; *Sme2.5_00670.1_g00012.1_DFR*, *Sme2.5_00747.1_g00013.1_AN11* expression level increased 3-4-fold and 7-10-fold under 38℃, respectively.~~ Under heat stress, *SmCHS4* and some anthocyanin biosynthesis related genes show different expression profiles at 38 ℃-3h (Fig 6),~~.~~ suggest that these co-up-regulated genes contribute to protect the eggplant at beginning of heat stress defense. In addition, 52 gene expression levels were reduced under heat stress, which was similar to Lv's results [7], while 35 gene expression levels were not identified. These results suggest that some key anthocyanin biosynthesis genes help to protect the eggplant from damage to heat stress. Moreover, these gene families exhibited two or more expression patterns and performed multiple genetic functions to regulate anthocyanin content. Combined with regulatory networks, it is possible to further understand the regulatory mechanism of peel color in eggplants.

~~**Fig 7. Key anthocyanin biosynthesis gene expression profiles in response to heat stress were observed at the highest expression level at 38℃-3h.**~~

Conclusions

In this study, a genome-wide analysis of the *SmCHS* gene family in eggplants was performed. The CHS protein biochemical characteristics, phylogenetic relationships, gene structures, *cis*-regulatory elements, regulatory network and functional predictions

of the *smCHS* gene family members were examined. The *SmCHS* gene family has conserved gene structure and functional diversification. CHS plays important roles in the anthocyanin biosynthesis pathway, exhibits two or more expression patterns and executes multiple functions to regulate anthocyanin content in eggplant peels under heat stress. The result of this study may contribute to the production of eggplant for further research on the functions, regulation and evolution of the CHS family.

Acknowledgments

This work was supported by the Agricultural Committee Basic Project (Shanghai Agricultural word (2015) No 6-2-3), the National Key Technology R&D Program during the 13th Five-Year Plan Period (2017YFD0101904) and the China Agriculture Research System (Grant No. CARS-25). The funding bodies did not play a role in the design of the study and the collection, analysis, and interpretation of data or in the composition of the manuscript.

Author contributions

DZ proposed the research, and ZZ and AD carried out the preparation and treatment of test materials. XL and SJ designed and performed the experiment. XW and SZ analyzed the data and wrote the manuscript. XW revised the article. All authors read and approved the final manuscript.

Supporting Information

Table S1. CHS protein sequences of Solanum species.

Table S2. Features of CHS genes identified in Solanum species.

Table S3. Primers used for real time PCR analysis.

Fig S1. Sequence alignment of all 73 CHS proteins of Solanum specie. Color bars on the left represent the 10 groups in Fig 4. Active site residues are highlighted in yellow, malony-CoA binding sites are highlighted in blue and other conserved sequence are shown in green.

Fig ~~S1~~S2. Interaction network key to anthocyanin biosynthesis in eggplant. The pink labels represent the CHS gene family.

References:

1. Dela G, Or E, Ovadia R, Nissim-Levi A, Weiss D, Oren-Shamir M. Changes in anthocyanin concentration and composition in 'Jaguar' rose flowers due to transient high-temperature conditions. Plant Sci. 2003; 164(3): 333-340. https://doi.org/10.1016/S0168-9452(02)00417-X.

2. Nozaki K, Takamura T, Fukai S. Effects of high temperature on flower colour and anthocyanin content in pink flower genotypes of greenhouse chrysanthemum (*Chrysanthemum morifolium* Ramat.). J Hortic Sci Biotechnol. 2006; 81(4): 728-734. https://doi.org/10.1080/14620316.2006.11512130.

3. Mori K, Goto-Yamamoto N, Kitayama M, Hashizume K. Loss of anthocyanins in red-wine grape under high temperature. J Exp Bot. 2007; 58(8): 1935-1945. https://doi.org/10.1093/jxb/erm055 PMID: 17452755.

4. Lin-Wang K, Micheletti D, Palmer J, Volz R, Lozano L, Espley R, et al. High temperature reduces apple fruit colour via modulation of the anthocyanin regulatory complex. Plant Cell Environ. 2011; 34(7): 1176-1190. https://doi.org/10.1111/j.1365-3040.2011.02316.x PMID: 21410713.

5. Movahed N, Pastore C, Cellini A, Allegro G, Valentini G, Zenoni S, et al. The grapevine VviPrx31 peroxidase as a candidate gene involved in anthocyanin degradation in ripening berries under high temperature. J Plant Res. 2016; 129(3): 513-526. https://doi.org/10.1007/s10265-016-0786-3 PMID: 26825649.

6. Pastore C, Dal Santo S, Zenoni S, Movahed N, Allegro G, Valentini G, et al. Whole plant temperature manipulation affects flavonoid metabolism and the transcriptome of grapevine berries. Front Plant Sci. 2017; 8: 929. https://doi.org/10.3389/fpls.2017.00929 PMID: 28634482.

7. Lv LL, Feng XF, Li W, Li K. High temperature reduces peel color in eggplant (*Solanum melongena*) as revealed by RNA-seq analysis. Genome. 2019; 62(7): 503-

512. https://doi.org/10.1139/gen-2019-0021 PMID: 31132325.

8. Dao T, Linthorst H, Verpoorte R. Chalcone synthase and its functions in plant resistance. Phytochem Rev. 2011; 10(3): 397. https://doi.org/DOI 10.1007/s11101-011-9211-7 PMID: 21909286.

9. Schröder J. A family of plant-specific polyketide synthases: facts and predictions. Trends Plant Sci. 1997; 2(10): 373-378. https://doi.org/10.1016/S1360-1385(97)01104-7.

10. Austin MB, Noel JP. The chalcone synthase superfamily of type III polyketide synthases. Nat Prod Rep. 2003; 20(1): 79-110. https://doi.org/10.1039/b100917f PMID: 12636085.

11. Winkel-Shirley B. It takes a garden. How work on diverse plant species has contributed to an understanding of flavonoid metabolism. Plant Physiol. 2001; 127(4): 1399-1404. https://doi.org/10.1104/pp.127.4.1399 PMID: 11743081.

12. Pang Y, Shen G, Wu W, Liu X, Lin J, Tan F, et al. Characterization and expression of chalcone synthase gene from *Ginkgo biloba*. Plant Sci. 2005; 168(6): 1525-1531. https://doi.org/10.1016/j.plantsci.2005.02.003 PMID: 24841790.

13. Winkel-Shirley B. Biosynthesis of flavonoids and effects of stress. Curr Opin plant Bio. 2002; 5(3): 218-223. https://doi.org/10.1016/S1369-5266(02)00256-X PMID: 11960739.

14. Buer CS, Imin N, Djordjevic MA. Flavonoids: new roles for old molecules. J Integr plant biol. 2010; 52(1): 98-111. https://doi.org/CNKI:SUN:ZWXB.0.2010-01-012 PMID: 20074144.

15. Falcone FM, Rius SP, Casati P. Flavonoids: biosynthesis, biological functions, and biotechnological applications. Front Plant Sci. 2012; 3: 222. https://doi.org/10.3389/fpls.2012.00222 PMID: 23060891.

16. Glagoleva AY, Ivanisenko NV, Khlestkina EK. Organization and evolution of the chalcone synthase gene family in bread wheat and relative species. BMC Genet. 2019; 20(1): 30. https://doi.org/10.1186/s12863-019-0727-y PMID: 30885129.

17. Chennupati P, Seguin P, Chamoun R, Jabaji S. Effects of high-temperature stress on soybean isoflavone concentration and expression of key genes involved in

isoflavone synthesis. J AgricFood Chem. 2012; 60(51): 12421-12427. https://doi.org/10.1021/jf3036319 PMID: 23199070.
18. Correia B, Rodriguez JL, Valledor L, Almeida T, Santos C, Cañal MJ, et al. Analysis of the expression of putative heat-stress related genes in relation to thermotolerance of cork oak. J Plant Physiol. 2014; 171(6): 399-406. https://doi.org/10.1016/j.jplph.2013.12.004 PMID: 24594391.
19. Liu R, Xu C, Zhang Q, Wang S, Fang W. Evolution of the chitin synthase gene family correlates with fungal morphogenesis and adaption to ecological niches. Sci Rep. 2017; 7: 44527. https://doi.org/10.1038/srep44527 PMID: 28300148.
20. Tropf S, Kärcher B, Schröder G, Schröder J. Reaction Mechanisms of Homodimeric Plant Polyketide Synthases (Stilbene and Chalcone Synthase). A single active site for the condensing reaction is sufficient for synthesis of stilbenes, chalcones, and 6'-deoxychalcones. J Biol Chem. 1995; 270(14): 7922-7928. https://doi.org/10.1074/jbc.270.14.7922 PMID: 7713888.
21. Ferrer J-L, Jez JM, Bowman ME, Dixon RA, Noel JP. Structure of chalcone synthase and the molecular basis of plant polyketide biosynthesis. Nat Struct Mol Biol. 1999; 6(8): 775. https://doi.org/10.1038/11553 PMID: 10426957.
22. Hideki H, Kenta S, Koji M, Tsukasa N, Satomi N, Akio O, et al. Draft genome sequence of eggplant (*Solanum melongena* L.): the representative *Solanum* species indigenous to the old world. DNA Res. 2014; 21(6): 649-660. https://doi.org/10.1093/dnares/dsu027 PMID: 25233906.
23. Consortium PGS. Genome sequence and analysis of the tuber crop potato. Nature. 2011; 475(7355): 189. https://doi.org/10.1038/nature10158 PMID: 21743474.
24. Consortium TTG. The tomato genome sequence provides insights into fleshy fruit evolution. Nature. 2012; 485(7400): 635. https://doi.org/10.1038/nature11119.
25. Schmidt MH-W, Vogel A, Denton AK, Istace B, Wormit A, van de Geest H, et al. De novo assembly of a new *Solanum pennellii* accession using nanopore sequencing. Plant Cell. 2017; 29(10): 2336-2348. https://doi.org/10.1105/tpc.17.00521 PMID: 29025960.
26. Kim S, Park M, Yeom S-I, Kim Y-M, Lee JM, Lee H-A, et al. Genome sequence

of the hot pepper provides insights into the evolution of pungency in *Capsicum* species. Nat Genet. 2014; 46(3): 270. https://doi.org/10.1038/ng.2877 PMID: 24441736.

27. Bombarely A, Moser M, Amrad A, Bao M, Bapaume L, Barry CS, et al. Insight into the evolution of the Solanaceae from the parental genomes of *Petunia hybrida*. Nat Plants. 2016; 2(6): 16074. https://doi.org/10.1038/nplants.2016.74 PMID: 27255838.

28. Sierro N, Battey JN, Ouadi S, Bakaher N, Bovet L, Willig A, et al. The tobacco genome sequence and its comparison with those of tomato and potato. Nat Commun. 2014; 5: 3833. https://doi.org/10.1038/ncomms4833 PMID: 24807620

29. Boratyn GM, Camacho C, Cooper PS, Coulouris G, Fong A, Ma N, et al. BLAST: a more efficient report with usability improvements. Nucleic Acids Res. 2013; 41(W1): W29-W33. https://doi.org/10.1093/nar/gkt282 PMID: 23609542.

30. Thompson JD, Gibson TJ, Plewniak F, Jeanmougin F, Higgins DG. The CLUSTAL_X windows interface: flexible strategies for multiple sequence alignment aided by quality analysis tools. Nucleic Acids Res. 1997; 25(24): 4876-4882. https://doi.org/10.1093/nar/25.24.4876 PMID: 9396791.

31. Tamura K, Stecher G, Peterson D, Filipski A, Kumar S. MEGA6: molecular evolutionary genetics analysis version 6.0. Mol Biol Evol. 2013; 30(12): 2725-2729. https://doi.org/10.1093/molbev/mst197 PMID: 24132122

32. Horton P, Park K-J, Obayashi T, Fujita N, Harada H, Adams-Collier C, et al. WoLF PSORT: protein localization predictor. Nucleic Acids Res. 2007; 35(suppl_2): W585-W587. https://doi.org/10.1093/nar/gkm259 PMID: 17517783.

33. Lescot M, Déhais P, Thijs G, Marchal K, Moreau Y, Van de Peer Y, et al. PlantCARE, a database of plant *cis*-acting regulatory elements and a portal to tools for *in silico* analysis of promoter sequences. Nucleic Acids Res. 2002; 30(1): 325-327. https://doi.org/10.1093/nar/30.1.325 PMID: 11752327.

34. Subramanian B, Gao S, Lercher MJ, Hu S, Chen W-H. Evolview v3: a webserver for visualization, annotation, and management of phylogenetic trees. Nucleic Acids Res. 2019. https://doi.org/10.1093/nar/gkz357 PMID: 31114888.

35. Zhang S, Zhang A, Wu X, Zhu Z, Yang Z, Zhu Y, et al. Transcriptome analysis revealed expression of genes related to anthocyanin biosynthesis in eggplant (*Solanum*

melongena L.) under high-temperature stress. BMC Plant Biol. 2019; 19(1): 1-13. https://doi.org/10.1186/s12870-019-1960-2 PMID: 31492114.

36. Chen C, Xia R, Chen H, He Y. TBtools, a Toolkit for Biologists integrating various biological data handling tools with a user-friendly interface. BioRxiv. 2018: 289660. https://doi.org/10.1101/289660.

37. Smoot ME, Ono K, Ruscheinski J, Wang P-L, Ideker T. Cytoscape 2.8: new features for data integration and network visualization. Bioinformatics. 2010; 27(3): 431-432. https://doi.org/10.1093/bioinformatics/btq675 PMID: 21149340.

38. Livak KJ, Schmittgen TD. Analysis of relative gene expression data using real-time quantitative PCR and the $2^{-\Delta\Delta CT}$ method. Methods. 2001; 25(4): 402-408. https://doi.org/10.1006/meth.2001.1262 PMID: 11846609.

39. Jacobs M, Rubery PH. Naturally occurring auxin transport regulators. Science. 1988; 241(4863): 346-349. https://doi.org/10.1126/science.241.4863.346 PMID: 17734864.

40. Brown DE, Rashotte AM, Murphy AS, Normanly J, Tague BW, Peer WA, et al. Flavonoids act as negative regulators of auxin transport in vivo in Arabidopsis. Plant Physiol. 2001; 126(2): 524-535. https://doi.org/10.2307/4279917 PMID: 11402184

41. Zhou B, Wang Y, Zhan Y, Li Y, Kawabata S. Chalcone synthase family genes have redundant roles in anthocyanin biosynthesis and in response to blue/UV A light in turnip (*Brassica rapa*; Brassicaceae). AM J Bot. 2013; 100(12): 2458-2467. https://doi.org/10.3732/ajb.1300305 PMID: 24197179.

42. Han Y, Ding T, Su B, Jiang H. Genome-wide identification, characterization and expression analysis of the chalcone synthase family in maize. Int J Mol Sci. 2016; 17(2): 161. https://doi.org/10.3390/ijms17020161 PMID: 26828478.

43. Han Y, Cao Y, Jiang H, Ding T. Genome-wide dissection of the chalcone synthase gene family in *Oryza sativa*. Mol Breeding. 2017; 37(10): 119. https://doi.org/10.1007/s11032-017-0721-x.

44. Zhang Y, Hu Z, Chu G, Huang C, Tian S, Zhao Z, et al. Anthocyanin accumulation and molecular analysis of anthocyanin biosynthesis-associated genes in eggplant (*Solanum melongena* L.). J Agr Food Chem. 2014; 62(13): 2906-2912.

https://doi.org/10.1021/jf404574c PMID: 24654563.

45. Jiang M, Liu Y, Ren L, Lian H, Chen H. Molecular cloning and characterization of anthocyanin biosynthesis genes in eggplant (*Solanum melongena* L.). Acta Physiol Plant. 2016; 38(7): 163. https://doi.org/10.1007/s11738-016-2172-0

46. Wu X, Zhang A, Zhu Z, Yao J, Zha D, Li X. Effects of High ÿtemperature Stress on Active Oxygen Metabolism, Anthocyanin Content and Its Main Synthases in Eggplant Peel. Acta Ag Jiangxi. 2018; 30(06): 1-5. https://doi.org/10.19386 j.cnki.jxnyxb.2018.06.01.

Dear Editors

Thank you and anonymous reviewers very much for your kind and useful comments for our manuscript "Genome-wide analysis of chalcone synthase (CHS) family from eggplant (*Solanum melongena* L.) in flavonoid biosynthetic pathway and expression pattern in response to heat stress" (PONE-D-19-33168). We have read and seriously considered the comments very carefully, checked and revised the manuscript many times. Major revisions throughout the revised manuscript have been made as followings.

1. The title name have been changed to "Chalcone synthase (CHS) family analysis from eggplant (*Solanum melongena* L.) in flavonoid biosynthetic pathway and expression pattern in response to heat stress"
2. The 'Introduction' section has been modified according to the comments
3. The results of all 73 CHS proteins sequence alignment, conserved residues and sequence diversity were analyzed.
4. We corrected some other grammatical errors and words spelling mistakes.

We would be grateful if you give us response soon.

Sincerely yours,

Xuexia Wu

The followings are responses to the comments point-by-point.

Review Comments to the Author

Reviewer #1: In this manuscript entitled 'Genome-wide analysis of chalcone synthase (CHS) family from eggplant (Solanum melongena L.) in flavonoid biosynthetic pathway and expression pattern in response to heat stress' the authors have identified CHS-encoding genes of eggplant, performed their in silico characterization and analysed the expression pattern of key genes associated with anthocyanin biosynthesis pathways. Here are the following comments on the research for the authors:

1. The manuscript need to be revised for its English, grammar and flow of information. For example in L130: 'The profiles of CHS (PF00195 and PF02797) were downloaded from the Pfam'- wwere need to be corrected to were. Line 198: 'the other 7 Solanaceae species ranged from 17.3 to 47.4, the length of protein ranged' kDa need to be added to the molecular weights mentioned here.

Answer: Thank you for pointing out the mistakes in the manuscript. We have corrected these issues in the revised manuscript according to comments, and checked the manuscript many times. Please refer to line 163 in revised manuscript with track changes.

2. It would be more informative for the readers if authors will provide a comparative mapping of CHS genes in the genome of other related plants where sequence information are available.

Answer: Chromosomal location of *CHS* genes were added in Table S1. Sequence alignment of all 73 CHS proteins of Solanum specie showed in the Fig S1. The Ⅷ, Ⅸ

and Ⅹ groups are distinguished from other groups mainly depends on the position 1-164 amino acids, Groups Ⅰ, Ⅱ and Ⅲ are relatively conservative at the position 260-360 amino acids, in which the other groups are very diverse(S1 Fig). Please refer to line 325-328.

3. Fig. 7. represents key anthocyanin biosynthesis gene expression profiles in response to heat stress. A bar diagram with proper error bars for individual gene will be the most appropriate way for presentation of this result. The authors are advised to prepare the same for readers convenient.

Answer: Thanks for your suggestion. Error bars have been added in the Fig7.

4. Authors have performed the phylogenetic classification of CHS gene in Solanaceae and found that 73 CHS genes have classified into 10 groups. However, it has not been mentioned or discussed that on what basis the CHS are classified and how one group differs with the others.

Answer: The results of all 73 CHS proteins sequence alignment were added in S1 Fig. Conserved residues and sequence diversity of 73 CHS proteins were analyzed. Please refer to line 240-244 and 325-328

5. As authors have mentioned in the text about some of the cis regulatory element present on all the SmCHS promoter, similarly they should mention the unique cis element for each SmCHS genes. Not all CHS genes had shown similar expression pattern, thus a probable correlation of cis regulatory element on promoter and expression pattern of the gene should be mentioned in the discussion.

Answer: I'm sorry we miss the point. We have added this to the 'result' section refer to line 303-307.

Reviewer #2: The manuscript entitled "Genome-wide analysis of chalcone synthase (CHS) family from eggplant (Solanum melongena L.) in flavonoid biosynthetic pathway and expression pattern in response to heat stress" by Wu et al. reports the in silico analysis of chalcone synthase (CHS) gene family in eggplant. In brief, the current manuscript presents biochemical characteristics, phylogenetic relationships, gene structures, cis-regulatory elements, regulatory network and functional predictions of the CHS gene family members.

The current quality of the manuscript leaves a number of questions to be answered, which are outlined below. The exposition of the results and its discussion in the manuscript needs a major revision including careful English usage and style edition.

1. The manuscript title starts with 'Genome-wide analysis'; however, most of the analysis has been performed on CHS proteins. The work performed in the manuscript does not justify the title.

Answer: We changed the manuscript title as to 'Chalcone synthase (CHS) family members analysis from eggplant (*Solanum melongena* L.) in the flavonoid biosynthetic pathway and expression patterns in response to heat stress'.

2. The ‘Introduction’ section of the manuscript is not coherent. Also, it does not describe the relevance of studying CHS gene family in proper manner.
Answer: We have added more information about CHS involve in synthesis of flavonoid compounds during heat stress defense. Please refer to line 70-80.

3. One of the major concerns is, authors have given RNA-Seq data in the result section. However, the source of this data is not described in the “Material and Method” section.
Answer: The overview of RNA-seq result from our lab was published (Zhang S et al, BMC Plant Biol. 2019 (1): 1-13). We therefore added a brief description of data source about RNA-seq in the “Material and Method” section. Please refer to line 196-198.

4. *Also, authors have not provided the proper statistics of the validation experiment. Statistical analysis should be done to evaluate the significance of qRT-PCR data. It is recommended to include statistical analysis in the ‘Materials and methods’ section.*
Answer: we have added the statistical analysis information of qRT-PCR in the ‘Materials and methods’ section. Please refer to line 223-225.

5. *Writing in Subheadings 4 to 7 under the ‘Results’ section is very technical. The data is not described properly. Authors have chosen to explain the dataset in the ‘Discussion’ section. The outcome of the experiments should be described in the ‘Results’ section and interpretation in consistence with the previous studies should be given in the ‘Discussion’ section.*
Answer: We agree and have revised the ‘results’ and ‘discussion’ accordingly. Please refer to line 351-361.

6. In Figure 3A, the abundance of cis-elements is given in all the CHS genes in eggplant. How the regulation of all the genes can be shown together?
Answer: We changed the histogram of figure 3A into a table to help readers understand the results.

7. In Figure 5, there is no downregulation value. How the data has been calculated? What control has been taken and how the data has been normalized?
Answer: Some errors have been corrected in line 351 of manuscript. Three *SmCHS* genes (*SmCHS1*, *SmCHS2*, and *SmCHS3*) were continuously downregulated under 38 ℃ and 45 ℃ treatment compared with the CK (**27 ℃**). Gene expression level was estimated by RNA-seq from mean FPKM (fragments per kilobase of exon model per million reads mapped) values for each treatment, and showed the expression patterns in heatmap. Each treatment had three biological replicates.

8. Figure 6, network is not very clear. A clear image has to be provided.
Answer: Is the network image you referred to Figure S2? We're sorry that there are too many genes in the network image. The picture can be expanded up to 6400%, so that the above words can be read clearly.

9. In ‘Material and Method’ section, what is CK? Why the samples harvested at 28º C are termed as CK and all the subsequent data is compared to CK?
Answer: The optimum growth temperature for eggplant is between 22 and 30°C. Eggplants subjected to high temperature may exhibit to stagnation of growth, abortion of flower buds, and decrease of pollen viability rate and fruit set, and the peel’s color will turn light when the temperature is over 35°C. Therefore, samples of 27 °C were termed as CK.

10. In Figure 7, all the genes are showing high expression at 3 h CK. Do authors have any explanation of these results? Also, this data has been recorded from which tissue?
Answer: Under heat stress, SmCHS4 and some anthocyanin biosynthesis related genes show different expression profiles at 38 ℃-3h. These results suggest that these co-up-regulated genes contribute to protect the eggplant at beginning of heat stress defense. These data were recorded from the eggplant peels, which is indicated in the Figure 5 and Figure 6.

11. *The other major issue is that this manuscript requires a thorough language editing since there are numerous grammatical errors including dropped articles, split infinitives, improper word usage etc. It is advisable that the manuscript must be edited by an English-speaking personal.*
Answer: We checked and revised the manuscript many times. Some grammatical errors and words spelling mistakes have been modified by English editing company of American Journal Experts (AJE).

12. *Reference section has to be rechecked. For example, in few places journal name is abbreviated (J Exp Bot) and in few places it is not (Plant physiology). In few references, journal name is capitalized and in others it is not.*
Answer: This issue has been corrected.

13. Biochemical analysis of CHS proteins (pI, molecular weight etc.) is not relevant to the manuscript. This data can be removed from the draft.
Overall, the manuscript is not of adequate quality. The manuscript should be revised thoroughly for data presentation, result interpretation, description and language.
Answer: Biochemical analysis of CHS proteins was removed.
