# Supplementary material for: Chalcone synthase (CHS) family members analysis from eggplant (Solanum melongena L.) in the flavonoid biosynthetic pathway and expression patterns in response to heat stress
Source: PLoS One. 2020 Apr 17;15(4):e0226537. doi: 10.1371/journal.pone.0226537 (PMC7164647; doi:10.1371/journal.pone.0226537)
Supplement: Attachment — Submitted filename: Response to Reviewers.docx [file pone.0226537.s006.docx]

Dear Editors

Thank you and anonymous reviewers very much for your kind and useful comments for our manuscript “Genome-wide analysis of chalcone synthase (CHS) family from eggplant (*Solanum melongena* L.) in flavonoid biosynthetic pathway and expression pattern in response to heat stress” (PONE-D-19-33168). We have read and seriously considered the comments very carefully, checked and revised the manuscript many times. Major revisions throughout the revised manuscript have been made as followings.

1. The title name have been changed to “Chalcone synthase (CHS) family analysis from eggplant (*Solanum melongena* L.) in flavonoid biosynthetic pathway and expression pattern in response to heat stress”

2. The ‘Introduction’ section has been modified according to the comments

3. The results of all 73 CHS proteins sequence alignment, conserved residues and sequence diversity were analyzed.

4. We corrected some other grammatical errors and words spelling mistakes.

We would be grateful if you give us response soon.

Sincerely yours,

Xuexia Wu

The followings are responses to the comments point-by-point.

Review Comments to the Author

*Reviewer #1: In this manuscript entitled ‘Genome-wide analysis of chalcone synthase (CHS) family from eggplant (Solanum melongena L.) in flavonoid biosynthetic pathway and expression pattern in response to heat stress’ the authors have identified CHS-encoding genes of eggplant, performed their in silico characterization and analysed the expression pattern of key genes associated with anthocyanin biosynthesis pathways. Here are the following comments on the research for the authors:*

*1. The manuscript need to be revised for its English, grammar and flow of information. For example in L130: ‘The profiles of CHS (PF00195 and PF02797) were downloaded from the Pfam’- wwere need to be corrected to were. Line 198: ‘the other 7 Solanaceae species ranged from 17.3 to 47.4, the length of protein ranged’ kDa need to be added to the molecular weights mentioned here.*

**Answer:** Thank you for pointing out the mistakes in the manuscript. We have corrected these issues in the revised manuscript according to comments, and checked the manuscript many times. Please refer to line 163 in revised manuscript with track changes.

*2. It would be more informative for the readers if authors will provide a comparative mapping of CHS genes in the genome of other related plants where sequence information are available.*

**Answer:** Chromosomal location of *CHS* genes were added in Table S1. Sequence alignment of all 73 CHS proteins of Solanum specie showed in the Fig S1. The Ⅷ, Ⅸ and Ⅹ groups are distinguished from other groups mainly depends on the position 1-164 amino acids, GroupsⅠ, Ⅱ and Ⅲ are relatively conservative at the position 260-360 amino acids, in which the other groups are very diverse(S1 Fig). Please refer to line 325-328.

*3. Fig. 7. represents key anthocyanin biosynthesis gene expression profiles in response to heat stress. A bar diagram with proper error bars for individual gene will be the most appropriate way for presentation of this result. The authors are advised to prepare the same for readers convenient.*

**Answer:** Thanks for your suggestion. Error bars have been added in the Fig7.

*4. Authors have performed the phylogenetic classification of CHS gene in Solanaceae and found that 73 CHS genes have classified into 10 groups. However, it has not been mentioned or discussed that on what basis the CHS are classified and how one group differs with the others.*

**Answer:** The results of all 73 CHS proteins sequence alignment were added in S1 Fig. Conserved residues and sequence diversity of 73 CHS proteins were analyzed. Please refer to line 240-244 and 325-328

*5. As authors have mentioned in the text about some of the cis regulatory element present on all the SmCHS promoter, similarly they should mention the unique cis element for each SmCHS genes. Not all CHS genes had shown similar expression pattern, thus a probable correlation of cis regulatory element on promoter and expression pattern of the gene should be mentioned in the discussion.*

**Answer:** I'm sorry we miss the point. We have added this to the ‘result’ section refer to line 303-307.

*Reviewer #2: The manuscript entitled “Genome-wide analysis of chalcone synthase (CHS) family from eggplant (Solanum melongena L.) in flavonoid biosynthetic pathway and expression pattern in response to heat stress” by Wu et al. reports the in silico analysis of chalcone synthase (CHS) gene family in eggplant. In brief, the current manuscript presents biochemical characteristics, phylogenetic relationships, gene structures, cis-regulatory elements, regulatory network and functional predictions of the CHS gene family members.*

*The current quality of the manuscript leaves a number of questions to be answered, which are outlined below. The exposition of the results and its discussion in the manuscript needs a major revision including careful English usage and style edition.*

*1. The manuscript title starts with ‘Genome-wide analysis’; however, most of the analysis has been performed on CHS proteins. The work performed in the manuscript does not justify the title.*

**Answer:** We changed the manuscript title as to ‘Chalcone synthase (CHS) family members analysis from eggplant (*Solanum melongena* L.) in the flavonoid biosynthetic pathway and expression patterns in response to heat stress’.

*2. The ‘Introduction’ section of the manuscript is not coherent. Also, it does not describe the relevance of studying CHS gene family in proper manner.*

**Answer:** We have added more information about CHS involve in synthesis of flavonoid compounds during heat stress defense. Please refer to line 70-80.

*3. One of the major concerns is, authors have given RNA-Seq data in the result section. However, the source of this data is not described in the “Material and Method” section.*

**Answer:** The overview of RNA-seq result from our lab was published (Zhang S et al, BMC Plant Biol. 2019 (1): 1-13). We therefore added a brief description of data source about RNA-seq in the“Material and Method” section. Please refer to line 196-198.

4. *Also, authors have not provided the proper statistics of the validation experiment. Statistical analysis should be done to evaluate the significance of qRT-PCR data. It is recommended to include statistical analysis in the ‘Materials and methods’ section.*

**Answer:** we have added the statistical analysis information of qRT-PCR in the ‘Materials and methods’ section. Please refer to line 223-225.

5. *Writing in Subheadings 4 to 7 under the ‘Results’ section is very technical. The data is not described properly. Authors have chosen to explain the dataset in the ‘Discussion’ section. The outcome of the experiments should be described in the ‘Results’ section and interpretation in consistence with the previous studies should be given in the ‘Discussion’ section.*

**Answer:** We agree and have revised the ‘results’ and ‘discussion’ accordingly. Please refer to line 351-361.

*6. In Figure 3A, the abundance of cis-elements is given in all the CHS genes in eggplant. How the regulation of all the genes can be shown together?*

**Answer:** We changed the histogram of figure 3A into a table to help readers understand the results.

*7. In Figure 5, there is no downregulation value. How the data has been calculated? What control has been taken and how the data has been normalized?*

**Answer:** Some errors have been corrected in line 351 of manuscript. Three *SmCHS* genes (*SmCHS1*, *SmCHS2*, and *SmCHS3*) were continuously downregulated under 38 ℃ and 45 ℃ treatment compared with the CK (**27 ℃**). Gene expression level was estimated by RNA-seq from mean FPKM (fragments per kilobase of exon model per million reads mapped) values for each treatment, and showed the expression patterns in heatmap. Each treatment had three biological replicates.

8. Figure 6, network is not very clear. A clear image has to be provided.

**Answer:** Is the network image you referred to Figure S2? We're sorry that there are too many genes in the network image. The picture can be expanded up to 6400%, so that the above words can be read clearly.

*9. In ‘Material and Method’ section, what is CK? Why the samples harvested at 28º C are termed as CK and all the subsequent data is compared to CK?*

**Answer:** The optimum growth temperature for eggplant is between 22 and 30°C. Eggplants subjected to high temperature may exhibit to stagnation of growth, abortion of flower buds, and decrease of pollen viability rate and fruit set, and the peel’s color will turn light when the temperature is over 35°C. Therefore, samples of 27 °C were termed as CK.

*10. In Figure 7, all the genes are showing high expression at 3 h CK. Do authors have any explanation of these results? Also, this data has been recorded from which tissue?*

**Answer:** Under heat stress, SmCHS4 and some anthocyanin biosynthesis related genes show different expression profiles at 38 ℃-3h. These results suggest that these co-up-regulated genes contribute to protect the eggplant at beginning of heat stress defense. These data were recorded from the eggplant peels, which is indicated in the Figure 5 and Figure 6.

11. *The other major issue is that this manuscript requires a thorough language editing since there are numerous grammatical errors including dropped articles, split infinitives, improper word usage etc. It is advisable that the manuscript must be edited by an English-speaking personal.*

**Answer:** We checked and revised the manuscript many times. Some grammatical errors and words spelling mistakes have been modified by English editing company of American Journal Experts (AJE).

12. *Reference section has to be rechecked. For example, in few places journal name is abbreviated (J Exp Bot) and in few places it is not (Plant physiology). In few references, journal name is capitalized and in others it is not.*

**Answer:** This issue has been corrected.

*13. Biochemical analysis of CHS proteins (pI, molecular weight etc.) is not relevant to the manuscript. This data can be removed from the draft.*

*Overall, the manuscript is not of adequate quality. The manuscript should be revised thoroughly for data presentation, result interpretation, description and language.*

**Answer:** Biochemical analysis of CHS proteins was removed.
