# Supplementary material for: Chalcone synthase (CHS) family members analysis from eggplant (Solanum melongena L.) in the flavonoid biosynthetic pathway and expression patterns in response to heat stress
Source: PLoS One. 2020 Apr 17;15(4):e0226537. doi: 10.1371/journal.pone.0226537 (PMC7164647; doi:10.1371/journal.pone.0226537)
Supplement: Attachment — Submitted filename: Review_March 2020.doc [file pone.0226537.s008.doc]

The manuscript entitled “Chalcone synthase (CHS) family member analysis from eggplant (*Solanum melongena* L.) in the flavonoid biosynthetic pathway and expression patterns in response to heat stress” by Wu et al. reports the *in silico* analysis of chalcone synthase (CHS) gene family followed by the expression analysis in eggplant.

First, I would appreciate the authors that they have addressed all the queries stated in the previous review. The quality of the manuscript has improved a lot. However, there are still few questions unanswered. Also, there are so many grammatical errors in the manuscript that altogether changes the meaning of the study. I have highlighted all the comments in the manuscript itself.

Please revise the manuscript once again for the grammatical corrections and other queries stated in the manuscript pdf file.
