# Supplementary material for: Chalcone synthase (CHS) family members analysis from eggplant (Solanum melongena L.) in the flavonoid biosynthetic pathway and expression patterns in response to heat stress
Source: PLoS One. 2020 Apr 17;15(4):e0226537. doi: 10.1371/journal.pone.0226537 (PMC7164647; doi:10.1371/journal.pone.0226537)
Supplement: Attachment — Submitted filename: Response to Reviewers.docx [file pone.0226537.s009.docx]

**Dear Editors,**

Thank you and anonymous reviewers very much for your kind and useful comments for our manuscript “Chalcone synthase (CHS) family members analysis from eggplant (*Solanum melongena* L.) in the flavonoid biosynthetic pathway and expression patterns in response to heat stress”. We have read and seriously considered the comments very carefully, checked and revised the manuscript many times. Major revisions throughout the revised manuscript have been made as followings.

1. We have revised all the errors according to the comments.

2. We have adjusted the order of the two subgraph (Fig3A and Fig3B) in Fig 3.

3. In the section of “mRNA regulatory network associated with anthocyanin biosynthesis in eggplant” of chapter Result, detail descriptions of relation between this section and SmCHS have added.

We would be grateful if you give us response soon.

Sincerely yours,

Xuexia Wu

The followings are responses to the comments point-by-point.

1. line 20. “isprimarily”

**Answer:** a space was added between the two words.

2. line 30-31. “occurrence of the duplicated words, “Under heat stress”

**Answer:** we removed duplicate words.

3. line 32. Wrong presentation of “at 38 ℃3h”

**Answer:** we have corrected.

4. line 33. “CHS protein biochemical characteristics”, deleted content in the result.

**Answer:** We have used evolutionary relationship instead of biochemical characteristics.

5. line 36-37. “SmCHS showed two or more expression patterns and performed multiple functions to regulate anthocyanin content. Combined with analysis of regulatory networks, to the results of this study may facilitate further research to understand the regulatory mechanism governing peel color in eggplants.”. Grammatical errors.

**Answer:** We have revised the sentences. We are sorry about our errors.

6. line 42-43. “subject to high”. Grammatical error.

**Answer:** We have corrected according to comments.

7. line 44. “decrease of pollen”.

**Answer:** We have revised it.

8. line 47. “molecular mechanism governing high temperature stress”

**Answer:** We have rewritten the sentence.

9. line 66. “for instance”

**Answer:** We have deleted the two words.

10. line 68-69. “in bread wheat”.

**Answer:** We have revised the manuscript.

11. line 72. Full-width comma is behind of the word “oak”.

**Answer:** We have revised it.

12. line 74. “*CHSV* and *CHSVII*”

**Answer:** We have changed italics to non-italics.

13. line 101-102. 'fruit shape has a' is not sounding proper.

**Answer:** We have revised the manuscript.

14. line 103. This line is not meant for the materials. It can be written into the Introduction section.

**Answer:** We have deleted the sentence.

15. line 141. “boostarp test”, Grammatical error.

**Answer:** We have used “Bootstarp value” instead of “bootstrap test”.

16. line 186. 1 μg of what? Please mention.

**Answer:** We have revised the manuscript according to the suggestion above.

17. Line 208. “The the length”, “the” repeated.

**Answer:** We have deleted the duplicate word according to the comment.

18. Line 209-210. Which other solanacae species?

**Answer:** We have added some details according to the comment.

19. Line212-214. What exactly as done after taking an average of the amino acids? It is still not clear

**Answer:** According the comment, we have rewritten the sentence.

20. Line 226-227. Is there any literature available citing this functional diversity for SmCHS 7?

**Answer:** We have added two references according the comment.

21. Line 250. Please replace 'and' with 'whereas'

**Answer:** We have corrected the error.

22. Line 252. 'cytoplasm' instead of 'cytoplasmic'

**Answer:** We have corrected the error.

23. Line 255 Table 2. 'Width' or "Length', please check once. Also, please add the unit. For example, in this case, it must be 'amino acids'

**Answer:** We have used “Length” instead of “Width” and have added the unit.

24. Line 257. Please describe Figure 3A first. Please try to maintain the sequence throughout the manuscript.

**Answer:** We have adjusted the order of the two subgraph (Fig3A and Fig3B) in Fig 3. we also have revised the manuscript.

25. Line 266-277. Please add 'element' after 'elicitor response'. Binding site or what? Please specify.

**Answer:** We have corrected them according the comments.

26. Line 293. Please add space before 'is'

**Answer:** We have revised the manuscript according the comment.

27. Line 300. Why SmCHS4 is in italics, but not the others. Moreover, the author is talking about protein sequences in this line. Do we really need to italicize it?

**Answer:** We have revised it.

28. Line 304. 'Colored' instead of 'color'

**Answer:** We have corrected the error.

29. Line 309. This RNAseq data was obtained from which tissue. Please specify.

**Answer:** We have added details of tissue.

30. Line 312. Why authors have chosen to write the name of these to genes in the list of genes not identified in the study?

**Answer:** We want to show that the CHS gene families may have tissue-specific or functionally differentiation. We have added this inference to the end of this paragraph.

31. Line 313-314. In this line, it is written that few of the SmCHSs were not identified. However, in the heatmap figure, expression data of all the CHS gene is given. How is that possible?

**Answer:** In order to show all genes related to anthocyanin biosythesis, the expression level of undetected genes were set to 0. These undetected genes were also shown in the heat map. We have rewritten these sentences.

32. Line 319. “at 45 ℃”

**Answer:** We have revised the manuscript.

33. Line 345. Please rewrite this line. It is not making any proper sense. Please add few lines in this paragraph to describe the link between this paragraph and SmCHSs.

**Answer:** We have added some details in this paragraph to describe the link between this paragraph and SmCHSs.

34. Line 350. The function can not be involved in anything. Please rewrite this line.

**Answer:** We have rewritten this sentence according the comment.

35. Line 398. Please capitalize 'biosynthesis'. You may write 'stress factors' instead of 'stressors'.

**Answer:** We have corrected the errors.

36. Line 405. Please cite the reference properly with the year.

**Answer:** We have corrected it.

37. Line 422. Please remove full stop.

**Answer:** We have removed the full stop.

38. Line 424. What is this reference? Please describe.

**Answer:** We have revised the manuscript.
